# A novel combination therapy for ER+ breast cancer suppresses drug resistance via an evolutionary double-bind

**DOI:** 10.1038/s44320-026-00191-z

**Published:** 2026-03-26

**Authors:** Rena Emond, Jeffrey West, Vince K Grolmusz, Patrick A Cosgrove, Aritro Nath, Alexander R A Anderson, Andrea H Bild

**Affiliations:** 1 City of Hope, Department of Medical Oncology and Therapeutics Research, Beckman Research Institute, City of Hope National Medical Center, Monrovia, 91016 CA USA; 2 Integrated Mathematical Oncology Dept. Moffitt Cancer Center, 12902 USF Magnolia Drive, Tampa, 33612 FL USA

**Keywords:** Disulfiram, Organoid, Chemotherapy Resistance, Evolutionary Game Theory, Mathematical Modeling, Cancer

## Abstract

Chemotherapy remains a commonly used and important treatment option for metastatic breast cancer. A majority of Estrogen Receptor-positive (ER + ) metastatic breast cancer patients ultimately develop resistance to chemotherapy, resulting in disease progression. We hypothesized that an “evolutionary double-bind”, where adapting to one treatment inadvertently makes cancer cells more susceptible to another treatment, would improve the effectiveness and durability of response to chemotherapy. This approach exploits vulnerabilities in acquired resistance mechanisms. Evolutionary models can be used to identify alternative treatment strategies that capitalize on such vulnerabilities in refractory cancers, leading to improved outcomes. To develop and test these models, ER+ breast cancer cell lineages sensitive and resistant to chemotherapy were grown in spheroids with varied initial population frequencies to measure cross-sensitivity and efficacy of chemotherapy and add-on treatments, such as disulfiram. Different treatment schedules were evaluated to identify the most effective strategy for reducing the selection of resistant populations, thereby preventing their proliferation and dominance. We developed a game-theoretic mathematical model, parameterized from this in vitro experimental data, and used it to predict the existence of a double-bind, where selection for resistance to chemotherapy induces sensitivity to disulfiram. The model predicts a dose-dependent re-sensitization to chemotherapy for monotherapy disulfiram.

## Synopsis

Evolutionary modeling of spheroids composed of sensitive and resistant cells show combination chemo+disulfiram treatment reduces resistance more than sequential therapy, predicting dose-dependent re-sensitization in vitro.

- Chemo-resistant ER⁺ breast cancer cells are vulnerable to disulfiram, exposing a collateral sensitivity absent in chemo-sensitive cells.
- Combined disulfiram + chemotherapy shows synergy in resistant lines, boosting efficacy beyond additive effects.
- Evolutionary game assays reveal cell-cell interactions sustain synergy even in mixed sensitive/resistant spheroids.
- A game-theoretic model predicts and experimental validation supports combo therapy best suppresses overall tumor fitness, outperforming sequential or alternating regimens.

## Abstract (web summary)

Evolutionary modeling of spheroids composed of sensitive and resistant cells show combination chemo+disulfiram treatment reduces resistance more than sequential therapy, predicting dose-dependent re-sensitization in vitro.

## Graphical abstract

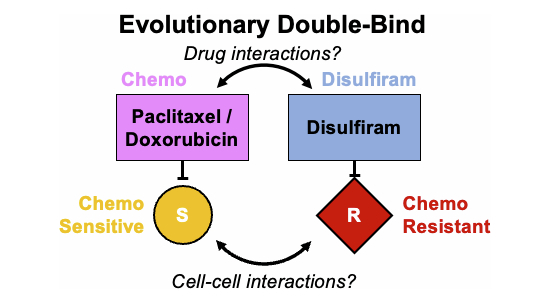

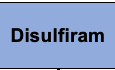

## Introduction

Identifying two drugs with synergistic action when combined is a promising strategy to combat drug resistance. While combination treatments have shown clinical promise (Wang and Minden, 2022), analytical methods to optimize treatment response remain limited due to heterogeneous response, multi-drug resistance, multiple resistance mechanisms, or simply a lack of available drugs that qualify as synergistic (Tufail et al, 2022; Ji et al, 2019). In this work, we implement a drug screening strategy in breast cancer that quantifies the presence of drug synergy in parental and evolved resistance breast cancer cell lines to develop new analysis methods for the assessment of combination therapy effectiveness. Importantly, this new analysis method also considers the potential for heterogeneous cell–cell interactions to negate (or promote) any drug synergy that is present. Here, we apply this screening process to identify effective chemotherapy add-on treatments in breast cancer cell lines. The goal of this drug screen is to identify a candidate drug that can be classified as an “evolutionary double-bind,” whereby the mechanism of resistance for a first-line therapy induces a vulnerability to this candidate second-line treatment (Basanta et al, 2011, 2012a; Gatenby et al, 2009a).

Drug combinations can be classified as additive (neutral), synergistic, or antagonistic. Identifying candidate drugs that are collaterally sensitive to first-line treatments represents a promising strategy to mitigate treatment resistance in antibiotics (Aulin et al, 2021; Nichol et al, 2019) and cancer (Dhawan et al, 2017). Collateral sensitivity is defined as the increased susceptibility to a particular drug when resistance to a separate drug has increased. Collateral sensitivity is often quantified using comprehensive multi-dose drug response assays, applying a range of monotherapy and combination dosing to characterize the synergy or antagonism between the two drugs using mathematical models (Meyer et al, 2020; Wooten et al, 2021; Meyer et al, 2019a). These approaches allow for rapid screening to identify drug candidates that may have beneficial efficacy in combination with first-line treatments. However, it is less common to repeat this process for evolved-resistance cell lines to note changes in collateral sensitivity after the onset of resistance. Changes in synergy after resistance or refractory disease remain an open question. Many published studies of drug screens focus on measuring drug-drug synergy only, but neglect analysis of (1) evolved resistance or (2) cell–cell interactions between parental and resistant lines.

Theoretical models indicate the potential for cell–cell interactions within heterogeneous tumors to either promote or negate drug synergy (Ma and Newton, 2021). While collateral sensitivity assays do provide insight into drug interactions that lead to increased efficacy when in combination, they do not address potential cell–cell interactions. For example, heterogeneous cell populations create the possibility for asymmetric interactions among cells that can alter responses to treatment (Neerven et al, 2021; Kim et al, 2022; Grolmusz et al, 2020; Freischel et al, 2021a; Wu et al, 2014a; Farrokhian et al, 2022; Paczkowski et al, 2021). Cells may interact through (1) competition for resources and space (Pelham et al, 2020), (2) cooperative production of resources (Marongiu et al, 2021), (3) mutualistic suppression of the immune response (Gourmet et al, 2024), or (4) inhibitory effects on neighboring healthy cells (Adler & Gordon, 2019). Thus, here we advocate for direct measurement of cell–cell interactions during the drug screening process to aid in determining the robustness of drug synergy within heterogeneous tumors. To measure cell–cell interactions, we employ the evolutionary game assay (EGA) (Kaznatcheev et al, 2019a) technique to quantify the frequency-dependent growth rates of cell lines in coculture under different treatments (Wu et al, 2014b; Farrokhian et al, 2022; Paczkowski et al, 2021; Kaznatcheev et al, 2019b). Evolutionary game theory has been extensively applied in cancer modeling to quantify cell–cell interactions between sensitive and resistant subpopulations (Stanková et al, 2018; Wölfl et al, 2022), for example, in non-small cell lung cancer (Kaznatcheev et al, 2019a) and breast cancer (Emond et al, 2023; Freischel et al, 2021b; Messan et al, 2021; Yang et al, 2022). However, the effect of cell–cell interactions on drug synergy remains an open question.

We hypothesized that an ideal secondary drug to combine with first-line therapy would possess the following characteristics. First, the existence of strong drug-drug synergistic effects. Second, the drug-drug synergy is maintained (or even strengthened) in the presence of evolved-resistance. Third, when co-culturing naive and evolved-resistance lines, any synergistic effects would again be maintained (or strengthened). Finally, the secondary drug would preferentially target evolved-resistance cells while simultaneously leading to total tumor regression. To reach these goals, the process for screening promising drugs must account wholistically for the underlying evolutionary and ecological dynamics at play within a heterogeneous, evolving system.

Once a candidate drug is identified, the question of drug scheduling (e.g., sequential or combination therapy) is addressed using mathematical modeling. Due to the nature of the evolutionary double-bind relying on the resistance mechanism of the first drug, the candidate second-line drug may be highly effective in the context of first-line resistance, but much less effective as a first-line treatment. It remains an open question whether two drugs in an evolutionary double-bind should be given in combination or in sequential therapeutic regimens. There has been recent interest in the development of treatment strategies which attempt to exploit competition between tumor subpopulations as a method of prolonging the emergence of resistance, often referred to as evolution-based treatment strategies (Gatenby et al, 2009b; Basanta et al, 2012b; Robertson-Tessi et al, 2023; Gatenby and Brown, 2020; Strobl et al, 2023). Historically, it has been a major challenge to parameterize mathematical models due to the lack of experimental characterization of relevant cell lines. Often, this has led to mathematical models relying heavily on untested assumptions about the proliferation cost of resistance, growth dynamics, plasticity, non-cell autonomous interactions, collateral sensitivity, and more. For example, many mathematical models take the assumption that the mechanism of resistance diverts energy and resources away from proliferation, resulting in a slower-growing resistant subpopulation. However, in some settings, resistant subpopulations may be associated with an increased proliferation (Wang et al, 2021). Similarly, mathematical models often implicitly assume either neutral or no competition between drug-sensitive and drug-resistant subpopulations. Thus, it is appropriate to design a drug screening process that quantifies these ecological and evolutionary dynamics under treatment.

### Screening for candidate evolutionary double-bind drugs in estrogen receptor-positive (ER+) breast cancer

Breast cancer is a leading cause of death worldwide. Breast cancer is classified into three main subtypes, with hormone receptor-positive tumors (including both estrogen and progesterone receptors) consisting of 75% of these cases (Malhotra et al, 2010). Roughly 20–40% of estrogen receptor-positive (ER+) patients eventually develop distant metastases and account for the majority of metastatic cancer cases (Stewart et al, 2003). First-line treatment for ER+ breast cancer is endocrine therapy. However, ~90% of all patients with metastatic breast cancer eventually develop resistance to endocrine therapy (Wood and Osborne, 1998 (EBCTCG, 2005). Chemotherapy is a treatment for advanced breast cancer that targets cell proliferation, and is considered a critical drug class in the metastatic setting; therefore, identifying strategies to optimize chemo-effectiveness is critical.

From a panel of several drug candidates, disulfiram is identified as a promising monotherapy to further test in combination with chemotherapy and assess potential treatment synergy. We hypothesize that disulfiram will aid in maintaining treatment sensitivity and allow for prolonged response for follow-up chemotherapy cycles. This study uses an in vitro 3D coculture model and evolutionary game-theoretic model to optimize treatment regimens in ER+ breast cancer cell spheroids consisting of resistant and sensitive cells, taking advantage of evolutionary dynamics and collateral sensitivity.

**Figure 1:**
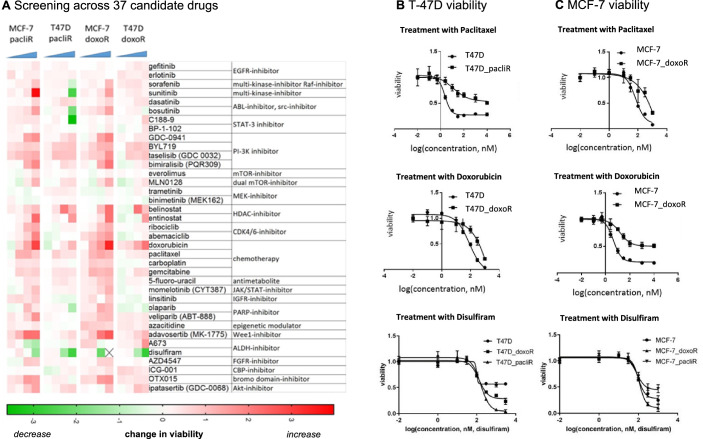
Candidate drugs screened for potential synergistic combinations. (**A**) Drug screen performed on MCF7 and T47D chemo-resistant cell lines (*n* = 4, technical). Thirty-seven candidate drugs of different classes of inhibitors, modulators, and chemotherapy were used across a range of treatment concentrations. The decrease in relative cell viability, normalized to the respective maternal sensitive cell line, is shown (green indicating highest decrease/effect in viability). (**B**) Growth curves (mean with SD shown for *n* = 3, technical) of T-47D sensitive and chemo-resistant cell populations treated with monotherapy paclitaxel, doxorubicin, or disulfiram at various concentrations. (**C**) Investigation repeated for MCF-7 cell lines. Source data are available online for this figure.

**Figure 2:**
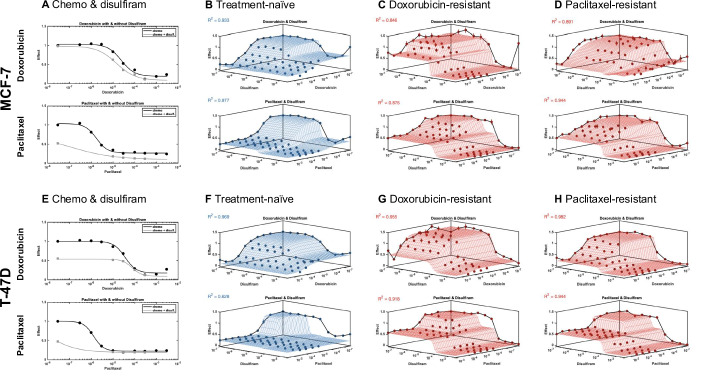
Dose response for MCF-7 and T-47D lines confronted to chemotherapy (doxorubicin and paclitaxel) in combination with disulfiram. Disulfiram investigation for synergistic potency in MCF-7 (**A**–**D**) and T47D (**E**–**H**); (*n* = 3, technical). (**A**,** E**) Dose response for chemo alone (black) and combination with disulfiram (gray). Drug concentrations are used in nM. (**B**,** G**) Combination dose response and synergy/antagonism is calculated using the MUSYC dose response framework (Wooten et al, 2021) for the treatment-naive line. Repeated for (**C**,** H**), doxorubicin evolved-resistant line and (**D**,** I**), paclitaxel evolved-resistant line. Synergistic potency (a_12_, a_21_) and efficacy (*β*) are shown in Table 1. Source data are available online for this figure.

We begin by screening for promising candidate drugs (Fig. 1), then quantify the synergy of the most promising candidate, disulfiram (Fig. 2; Table 1). Next, we perform an evolutionary game assay to quantify cell–cell competition across treatment conditions. An integrative mathematical-experimental analysis predicts that a high dose combination of both drugs (chemotherapy and disulfiram) in tandem maximally suppresses both resistant and treatment-naive cells. Finally, we validate the approach by considering alternating (sequential) therapy strategies, which underperform combination treatment, as predicted. In our assessment of disulfiram as a candidate combination therapy with chemotherapy, we classified it as an evolutionary double-bind due to confirming (1) drug-drug synergy between the two therapies and (2) that disulfiram selectively creates a fitness disadvantage for resistant cells, especially when in competition with sensitive cells. Taken together, these two metrics provide empirical evidence that the disulfiram-chemotherapy combination therapy qualifies as an evolutionary double-bind.

**Table 1:** Combination drug potency and efficacy values.

Cell line	Drug 1	Drug 2	Type	Batch	Potency	Log(a_21_)	Efficacy	*β*
MCF7	Doxo	Disu	Naive	1	Neutral	**0.03** (−0.27, 0.36)	**Synergy**	**0.05** (0.04, 0.19)
				2	Neutral	**−0.01** (−0.29, 0.29)	Neutral	**−0.22** (−0.89, 0.22)
				3	Neutral	**−0.14** (−0.46, 0.15)	**Synergy**	**0.07** (0.04, 0.22)
				4	Neutral	**0.06** (−0.32, 0.35)	**Synergy**	**0.05** (0.01, 0.19)
MCF7	Doxo	Disu	DoxoR	1	Neutral	**2.6** (−0.18, 4.76)	**Synergy**	**0.34** (0.24, 0.82)
				2	**Synergy**	**1.5** (0.39, 2.73)	**Synergy**	**0.29** (0.2, 0.6)
				3	**Synergy**	**1.1** (0.53, 2.18)	**Synergy**	**0.44** (0.32, 0.8)
				4	**Synergy**	**4.2** (1.00, 7.06)	**Synergy**	**0.46** (0.32, 1.01)
MCF7	Doxo	Disu	PaclR	1	Neutral	**1.1** (−0.62, 2.53)	**Synergy**	**0.19** (0.08, 0.41)
				2	**Synergy**	**1.4** (0.05, 2.66)	**Synergy**	**0.19** (0.11, 0.48)
				3	Neutral	**0.8** (−0.14, 2.11)	Neutral	**−0.23** (−0.93, 0.3)
				4	**Synergy**	**1.8** (0.78, 3.76)	**Antagonism**	**−0.28** (−0.33, −0.06)
MCF7	Pacl	Disu	Naive	1	Neutral	**−0.2** (−0.56, 0.69)	**Synergy**	**0.19** (0.17, 0.22)
				2	Neutral	**0.5** (−0.55, 3.26)	**Synergy**	**0.15** (0.13, 0.19)
				3	Neutral	**−0.2** (−0.62, 0.51)	**Synergy**	**0.15** (0.13, 0.2)
				4	Neutral	**−0.1** (−0.61, 0.6)	**Synergy**	**0.18** (0.16, 0.26)
MCF7	Pacl	Disu	PaclR	1	Neutral	**4.6** (−0.17, 4.59)	Synergy	**0.08** (0.05, 0.21)
				2	Neutral	**−0.6** (−4.62, 3.38)	Neutral	**0.06** (−0.03, 0.19)
				3	Neutral	**1.8** (−0.18, 5.75)	**Synergy**	**0.21** (0.14, 0.45)
				4	Neutral	**6.8** (0.00, 8.00)	Neutral	**0.03** (0.00, 0.13)
MCF7	Pacl	Disu	DoxoR	1	Neutral	**−6.6** (−8.00, 0.00)	Neutral	**0.01** (−0.14, 0.07)
				2	Neutral	**0.8** (−0.56, 1.39)	Neutral	**0.00** (−0.02, 0.08)
				3	Neutral	**0.0** (−8, 0.00)	**Synergy**	**0.06** (0.03, 0.1)
				4	Neutral	**7.0** (0, 7.98)	Neutral	**−0.01** (−0.02, 0.08)
T-47D	Doxo	Disu	Naive	1	Neutral	**−0.2** (−0.44, 0.06)	Neutral	**−0.34** (−0.48, 0.11)
				2	Neutral	**−0.3** (−0.53, 0.11)	Neutral	**−0.16** (−0.98, 0.14)
				3	Neutral	**−0.3** (−0.57, 0.02)	Neutral	**0.10** (−0.99, 0.16)
				4	Neutral	**−0.3** (−0.60, 0.20)	Neutral	**−0.09** (−0.96, 0.13)
T-47D	Doxo	Disu	DoxoR	1	Neutral	**0.5** (−0.18, 1.47)	**Synergy**	**0.10** (0.04, 0.31)
				2	Neutral	**0.3** (−0.32, 1.24)	**Synergy**	**0.11** (0.07, 0.26)
				3	Neutral	**−0.2** (−3.96, 1.09)	**Synergy**	**0.21** (0.14, 0.29)
				4	Neutral	**−0.1** (−4.12, 1.38)	**Synergy**	**0.12** (0.03, 0.21)
T-47D	Doxo	Disu	PaclR	1	Neutral	**0.7** (−0.5, 1.82)	Neutral	**0.02** (−0.01, 0.09)
				2	Neutral	**−1.1** (−5.1, 0.71)	Neutral	**0.05** (−0.03, 0.09)
				3	Neutral	**0.3** (−0.31, 0.86)	**Synergy**	**0.03** (0.02, 0.08)
				4	**Synergy**	**0.7** (0.15, 1.26)	**Synergy**	**0.04** (0.02, 0.1)
T-47D	Pacl	Disu	Naive	1	**Antagonism**	**−0.5** (−0.82, −0.13)	**Synergy**	**0.05** (0.05, 0.08)
				2	Neutral	**−0.5** (−0.9, 0.24)	**Synergy**	**0.04** (0.03, 0.06)
				3	Neutral	**−0.3** (−0.68, 0.64)	**Synergy**	**0.03** (0.02, 0.06)
				4	Neutral	**4.0** (0.00, 8.00)	**Synergy**	**0.05** (0.05, 0.08)
T-47D	Pacl	Disu	PaclR	1	Neutral	**−1.3** (−5.22, 0.9)	Neutral	−**0.01** (−0.03, 0.04)
				2	Neutral	**8.0** (0.00, 8.00)	Neutral	**0.01** (0.00, 0.06)
				3	Neutral	**8.0** (0.00, 8.00)	Neutral	**0.00** (−0.01, 0.05)
				4	Neutral	**0.5** (−3.15, 4.85)	Neutral	**0.00** (−0.01, 0.07)
T-47D	Pacl	Disu	DoxoR	1	Neutral	**−0.07** (−4.03, 2.16)	**Synergy**	**0.03** (0.02, 0.1)
				2	**Synergy**	**6.6** (0.8, 6.35)	Neutral	**0.00** (−0.01, 0.07)
				3	**Synergy**	**0.8** (0.38, 1.27)	Neutral	**0.01** (−0.01, 0.07)
				4	**Synergy**	**2.6** (0.9, 4.94)	Neutral	**0.01** (−0.02, 0.07)

Quantification of drug synergy for two cell lines (MCF7, T-47D) when confronted with two drugs: chemotherapy (paclitaxel or doxorubicin) in combination with disulfiram. Cell lines are either naive, or evolved resistance to paclitaxel or doxorubicin. Each dose response checkerboard assay is repeated four times (batch numbers 1–4 are related to different concentrations of disulfiram, in increasing concentrations), and synergy is measured using the MUSYC software package (Wooten et al, 2021; Meyer et al, 2019a). We consider the effect of disulfiram on the EC50 (potency; log(a_21_): see Methods) and on maximal response (efficacy; *β*: see Methods).

## Results

### Candidate drugs screened for potential synergistic combinations

Thirty-seven candidate drugs were screened to determine the potential for synergistic combinations with chemotherapy across two cell lines (MCF-7, T-47D) with evolved resistance to two different chemotherapy drugs (paclitaxel and doxorubicin). The heatmap in Fig. 1A, indicates potential collaterally sensitivity drugs (green boxes) which improve response in resistant lines. The panel of drugs chosen included several therapies, either FDA-approved for oncological use or tested in clinical trials. These include known targeted therapies such as EGFR, PIK3CA/AKT/mTOR, HDAC, CDK 4/6 inhibitors (among others), chemotherapy, and other drugs showing promising off-label use in various oncological settings (Moo et al, 2018; Zhong et al, 2021). A range of increasing drug concentrations (indicated across the columns from left to right) were used to determine the relative viability between resistant and sensitive lines. Candidates with strong drug effect on chemo-resistant cell lines, represented by a decrease in relative cell viability normalized to the maternal sensitive cell line, are shown in green. Disulfiram (an ALDH inhibitor) demonstrated a dose-dependent increased sensitivity in resistant cells across all four across all four chemo-resistant cell line pairs identifying it as a potential candidate for synergy across chemo-resistant lines. Follow-up dose-response experiments in sensitive and doxorubicin- and paclitaxel-resistant cell lines confirmed both resistance to mono-chemotherapy as well as the acquired sensitivity to disulfiram in resistant lines compared to the parental sensitive line (Fig. 1B,C). In addition, chemo-resistant cells exhibited decreased viability when confronted with disulfiram treatment in a concentration-dependent manner. While this was observed in both T47D and MCF7 chemo-resistant cell lines, there were differences in sensitivity to disulfiram between the specific chemo-resistant lines. For example, in T47D, paclitaxel resistant cells harbored a greater sensitivity to disulfiram compared to doxorubicin resistant cells (Fig. 1B). Whereas, MCF7 doxorubicin resistant cells were more negatively affected by disulfiram compared to the paclitaxel resistant line (Fig. 1C). This suggests the molecular resistance mechanisms arising from different chemo-resistant specific phenotypes influence the degree of disulfiram’s targeted drug effect. The range of drug response between the cell lines highlights the importance of concentration considerations during treatment and across different resistant profiles. These findings from our comprehensive drug screen suggest disulfiram as a favorable lead candidate for potential synergistic combination treatment with chemotherapy.

### Quantifying collateral sensitivity

After disulfiram was shown to improve response in chemo-resistant lines, potential synergistic combination treatment with either paclitaxel or doxorubicin was next explored. T-47D and MCF-7 sensitive and resistant lines were confronted with a combination treatment of disulfiram with chemotherapy at various monotherapy and combination concentrations, and the drug effect was assessed by a measure of decreased cell viability. An observed increase in efficacy was seen in both lines when combining chemotherapy with disulfiram treatment compared to mono- chemotherapy (Fig. 2A,E). However, with a simple one-dimensional dose response curve, it is not known whether this increase is strictly additive, or if there exist synergistic or antagonistic effects between the two drugs.

To assess and quantify synergy (or antagonism), we performed dose-response assays for MCF-7 and T-47D sensitive lines, treated with either doxorubicin or paclitaxel and disulfiram (Fig. 2B,F), as well as MCF-7 and T47D chemo-resistant lines, treated with each chemotherapy and disulfiram (Fig. 2C,D,G,H). This data were subsequently fed into the multi-dimensional synergy of combinations (MUSYC) framework (Wooten et al, 2021). MUSYC quantifies two types of synergy: synergistic potency (fold-change in the EC50 half-max concentration when adding the drug) and synergistic efficacy (percent decrease in max effect at high concentrations of both drugs) (Meyer et al, 2020, 2019a). A full description of drug synergy is shown in Table 1. In treatment-naive lines, the chemo-disulfiram combination is typically neutral (neither synergistic nor antagonistic) in efficacy and potency, suggesting an additive effect that exists between the drugs. This neutrality remains across most evolved-resistant cell lines, with the exception of doxorubicin-resistant MCF-7 cells (Fig. 2C) which shows synergistic potency between doxorubicin and disulfiram.

This analysis indicates that disulfiram is an effective drug in combination with chemotherapy strictly due to additive (or at best, weakly synergistic) effects. Importantly, we have confirmed that additivity is maintained even after evolved resistance (Fig. 2C,D,G,H). It’s also important to note that the combination (high dose of both chemotherapy and disulfiram) results in maximum efficacy across all cell lines, chemotherapies, and resistance settings.

**Figure EV1:**
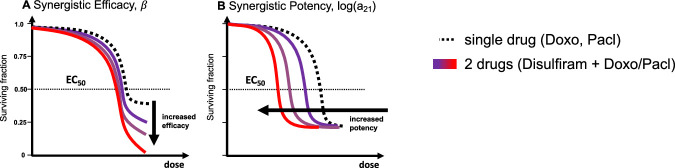
Schematic representation of MUSYC parameters describing synergistic potency and synergistic efficacy. (**A**) Synergistic efficacy, *β*, leads to a decrease in surviving fraction at high doses when the second drug is delivered in combination. (**B**) Synergistic potency, log(a_21_), leads to a decrease in the EC_50_ concentration when the second drug is delivered in combination.

Interestingly, chemotherapy has an additional stabilizing effect on disulfiram. Visually, disulfiram has a non-monotonic drug effect at high doses when given in isolation (Fig. 2B–D,F). While intermediate doses (e.g., 10^−4^, 10^−5^) achieve increasing response, higher doses (e.g., 10^−3^) of disulfiram result in less response, causing the dose response curve to be non-monotonic. This observed effect disappears when disulfiram is given in combination with chemotherapy. Since the MUSYC framework assumes a monotonic effect, it cannot account for this stabilization effect (Fig. EV1A,B). This analysis ignores any cell–cell interactions between chemo-resistant lines and treatment-naive lines, which we consider in the next section.

### Quantifying cell–cell interactions

Thus far, we have considered treatment-naive cell lines and resistant cell lines in isolation. Next, we perform the Evolutionary Game Assay (Kaznatcheev et al, 2019a) as a method for quantifying the fitness of cell lines in coculture and under different therapies. Here, we define fitness to be synonymous with the growth rate of a cell line. We quantify changes in growth rate as a function of the frequency of competing cell types, known as frequency-dependent growth dynamics.

**Figure EV2:**
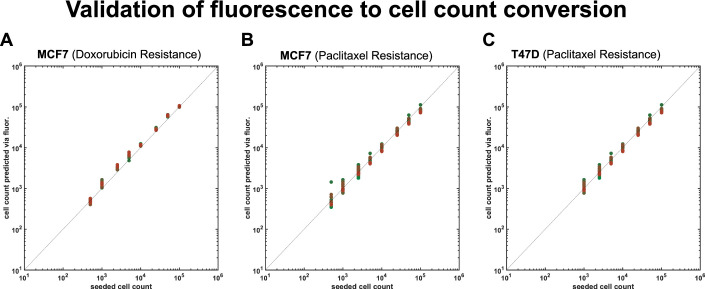
Validation of fluorescence intensity, F, to cell count, N, equation (see Methods). (**A**) Fluorescence to cell count conversion for MCF7 (Doxorubicin resistance). (**B**) Fluorescence to cell count conversion for MCF7 (Paclitaxel-resistance). (**C**) Fluorescence to cell count conversion for T47D (Paclitaxel-resistance). Known values of cells seeded (x-axis) are compared to predicted cell counts based on measured fluorescence intensity (y-axis). The model provides accurate cell count predictions, falling on the unity line (black line) across all initial sensitive-to-resistant ratios (color).

**Figure 3:**
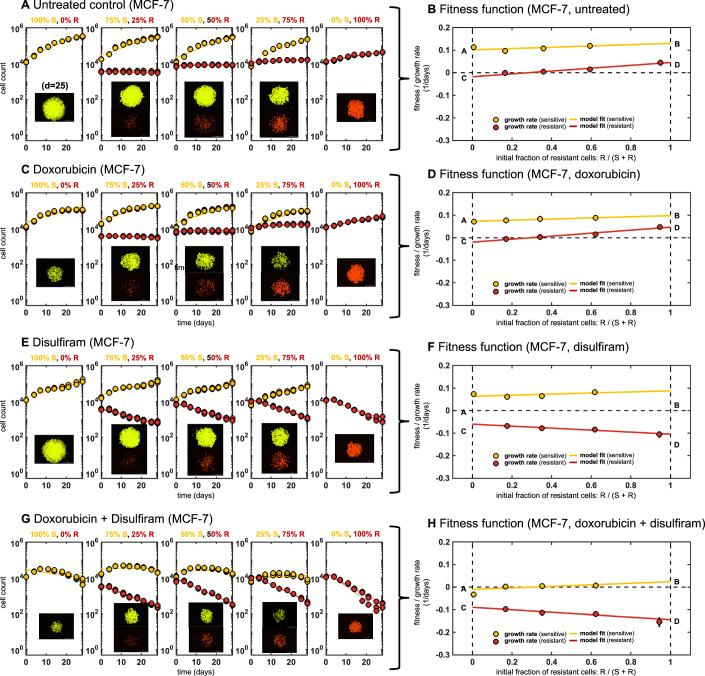
Evolutionary game assay. MCF7 cells are seeded with a ratio of resistant to naive of 0%R, 25%R, 50%R, 75%R, and 100%R. (**A**) The best fit exponential growth rate is measured for each replicate (*n* = 3, technical), repeated for each ratio. (**B**) These growth rates (mean and standard deviation shown for three replicates at each ratio) determine the cell type’s fitness as a function of resistant fraction. A linear fitness function is fit with parameters A, B, C, D denoted (See Methods). The process is repeated for spheroids treated with (**C**,** D**), doxorubicin, (**E**,** F**), disulfiram and (**G**,** H**), doxorubicin and disulfiram combination therapy. Images shown at day 25. Source data are available online for this figure.

By labeling the resistant and sensitive cell lines with different fluorescent proteins, this in vitro model allowed for the analysis of each cell lines’ different proliferative dynamics and competition in a 3D coculture system using imaging and fluorescence intensity tracking relative to growth (Fig. EV2A–C). The 3D cell culture allows cells to create cell–cell interactions which resembles more closely the in vivo ecosystem, therefore results obtained from 3D cell culture are clinically more relevant (Manduca et al, 2023). Cells are seeded across a range of initial resistant (chemotherapy-resistant, R) to naive (treatment-sensitive cells, S) ratios: 0%R, 25%R, 50%R, 75%R, and 100%R. The exponential growth rate is measured for each initial condition, and repeated for untreated control, doxorubicin, disulfiram, and combination doxorubicin and disulfiram (Fig. 3A,C,E,G, respectively). The collection of experiments gives the cell fitness (e.g., growth rate) as a function of the fraction of resistant cells (Fig. 3B,D,F,H).

In untreated conditions, naive cells tend to grow faster across all initial conditions, suggesting a cost (i.e., a reduction in growth rate) to the mechanism of resistance to chemotherapy (Fig. 3A). Naive cells maintain a higher fitness in untreated conditions, a result of a higher proliferation rate, and a competitive advantage (Fig. 3B). Somewhat unsurprisingly, chemotherapy decreases the fitness of naive cells to a greater extent than resistant cells (Fig. 3C), across all initial resistant fractions (Fig. 3D). As expected, chemo-resistant cells are better adept to withstand the effects of chemotherapy compared to sensitive cells. In contrast, the introduction of disulfiram decreases the fitness of resistant cells to a greater extent (Fig. 3E), across all fractions (Fig. 3F). When given in combination, chemotherapy and disulfiram together negatively impact the fitness of both populations in coculture (Fig. 3G), and the additive effect of both drugs decreases the overall total cell count (Fig. 3H).

Growth dynamics can be classified as having frequency-dependence if the fitness function changes with the seeded ratio (frequency) of resistant cells. Data for each coculture ratio is fit using a linear function (solid red, yellow lines in Fig. 3B,D,F,H). The fitness functions shown in Fig. 3 are characterized by two parameters for each cell line (four total parameters): growth rate for monoculture 100% sensitive and 100% resistant (dashed lines in Fig. 3B,D,F,H). Fitness (growth rate) is a linear function between the monoculture growth rates for sensitive (labeled A, B) and resistant lines (labeled C, D). These four parameters comprise the payoff matrix (see Methods), providing a complete characterization of the fitness function of two competing cell lines. Thus, we define two important quantities that determine the selection dynamics in this model system: ${\Delta }_{S}$ and ${\Delta }_{R}$.

$${\Delta }_{S}=B-D$$

and

$${\Delta }_{R}=C-A$$

These two quantities represent the invasion fitness for each cell type S, R, when the prevalence of that cell type is small. For example, if ${\Delta }_{R} > 0$, resistant cells are expected to invade a population of sensitive cells. Whether that invasion continues to full dominance (resistant cells fully take over) depends on the value of ${\Delta }_{S}$ (see Methods).

### Classifying selection dynamics

**Figure 4:**
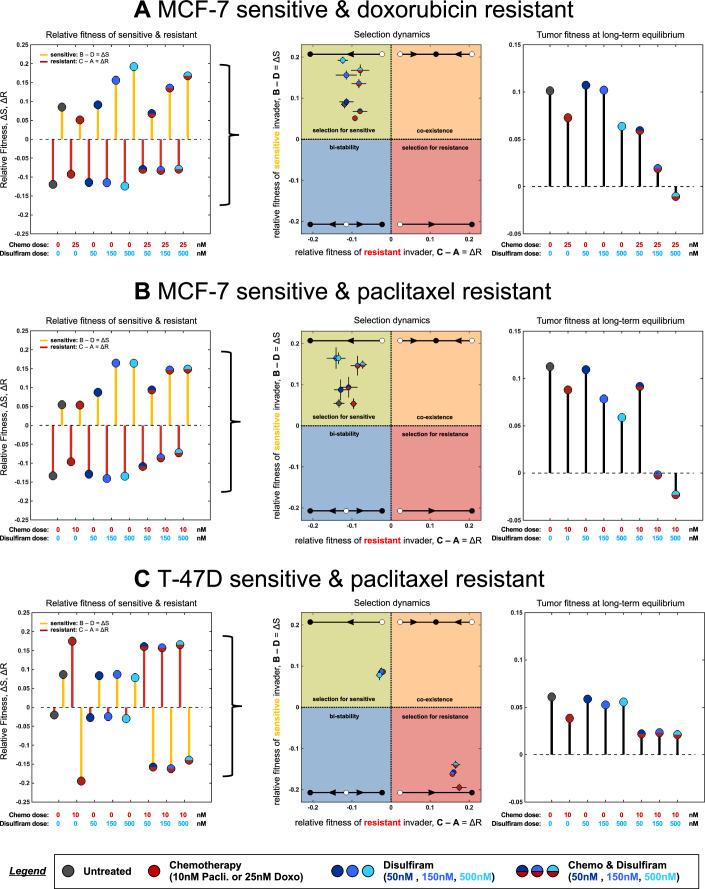
Fitness parameters determine selection dynamics. (**A**) MCF7 sensitive and doxorubicin-resistant lines (mean and standard deviation shown for *n* = 3, technical), Left (relative fitness): fitness functions fit in Fig. 3, shown across all monotherapy or combination therapy options. Relative fitness of sensitive (parameters B - D) and resistant (parameters C - A) shown. Middle (selection dynamics): relative fitness plotted in a quadrant showing selection dynamics as a function of B - D and C- A. All treatment scenarios are selected for sensitivity to varying degrees. Right (tumor fitness): overall tumor fitness of mixed populations shown (long-term model prediction; see Methods). High-dose combination therapy minimizes tumor fitness. (**B**) relative fitness, selection dynamics, and tumor fitness are shown for MCF7 sensitive and paclitaxel-resistant cell lines; (mean and standard deviation shown for *n* = 3, technical). (**C**) relative fitness, selection dynamics, and tumor fitness are shown for T47D sensitive and paclitaxel-resistant cell lines; (mean and standard deviation shown for *n* = 3, technical). Source data are available online for this figure.

**Figure EV3:**
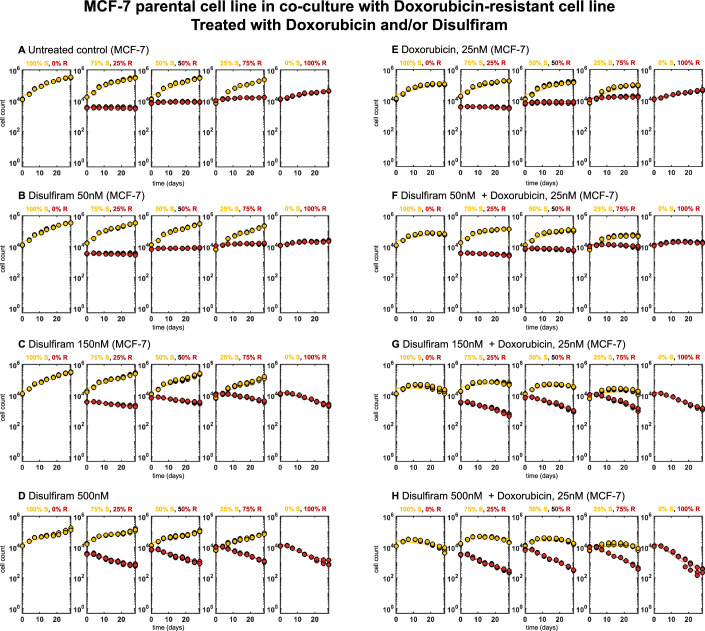
Evolutionary game assay: Cells are seeded with a ratio of resistant to naive of 0%R, 25%R, 50%R, 75%R, and 100%R across all treatment conditions. The best fit exponential growth rate is measured for each replicate (*n* = 3), repeated for each ratio. (**A**) Untreated control (MCF7). (**B**) Disulfiram, 50 nM (MCF7). (**C**) Disulfiram, 150 nM (MCF7). (**D**) Disulfiram, 500 nM (MCF7). (**E**) Doxorubicin, 25 nM (MCF7). (**F**) Disulfiram, 50 nM + Doxorubicin, 25 nM (MCF7). **G**) Disulfiram, 150 nM + Doxorubicin, 25 nM (MCF7). (**H**) Disulfiram, 500 nM + Doxorubicin, 25 nM (MCF7).

**Figure EV4:**
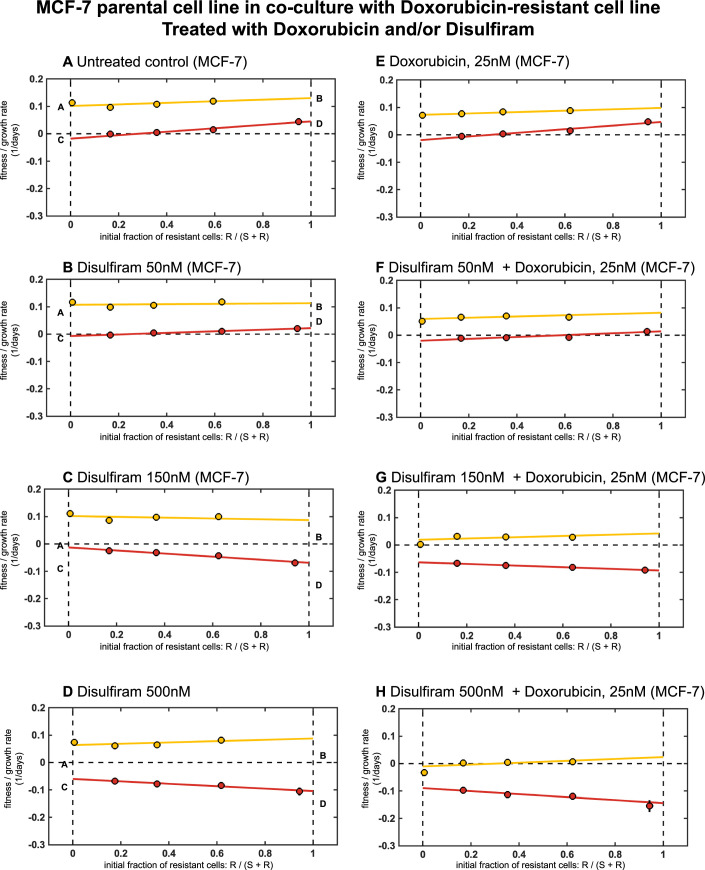
Growth rates (mean and standard deviation shown for 3 replicates at each ratio) for the corresponding coculture experiments in Fig. EV3 determine the cell type’s fitness as a function of resistant fraction. A linear fitness function is fit with parameters A, B, C, D denoted (See Methods). Shown for MCF-7 parental cell line in coculture with Doxorubicin-resistant cell line, treated with Doxorubicin and/or Disulfiram as indicated. (**A**) Untreated control (MCF7). (**B**) Disulfiram, 50 nM (MCF7). (**C**) Disulfiram, 150 nM (MCF7). (**D**) Disulfiram, 500 nM (MCF7). (**E**) Doxorubicin, 25 nM (MCF7). (**F**) Disulfiram, 50 nM + Doxorubicin, 25 nM (MCF7). (**G**) Disulfiram, 150 nM + Doxorubicin, 25 nM (MCF7). (**H**) Disulfiram, 500 nM + Doxorubicin, 25 nM (MCF7).

**Figure EV5:**
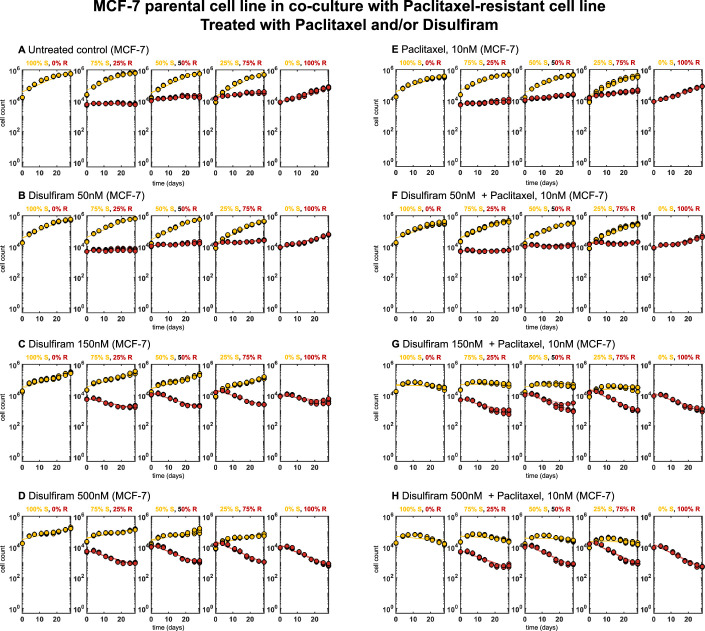
Evolutionary game assay: Cells are seeded with a ratio of resistant to naive of 0%R, 25%R, 50%R, 75%R, and 100%R across all treatment conditions. The best fit exponential growth rate is measured for each replicate (*n* = 3), repeated for each ratio. (**A**) Untreated control (MCF7). (**B**) Disulfiram, 50 nM (MCF7). (**C**) Disulfiram, 150 nM (MCF7). (**D**) Disulfiram, 500 nM (MCF7). (**E**) Paclitaxel, 10 nM (MCF7). (**F**) Disulfiram, 50 nM + Paclitaxel, 10 nM (MCF7). (**G**) Disulfiram, 150 nM + Paclitaxel, 10 nM (MCF7). (**H**) Disulfiram, 500 nM + Paclitaxel, 10 nM (MCF7).

**Figure EV6:**
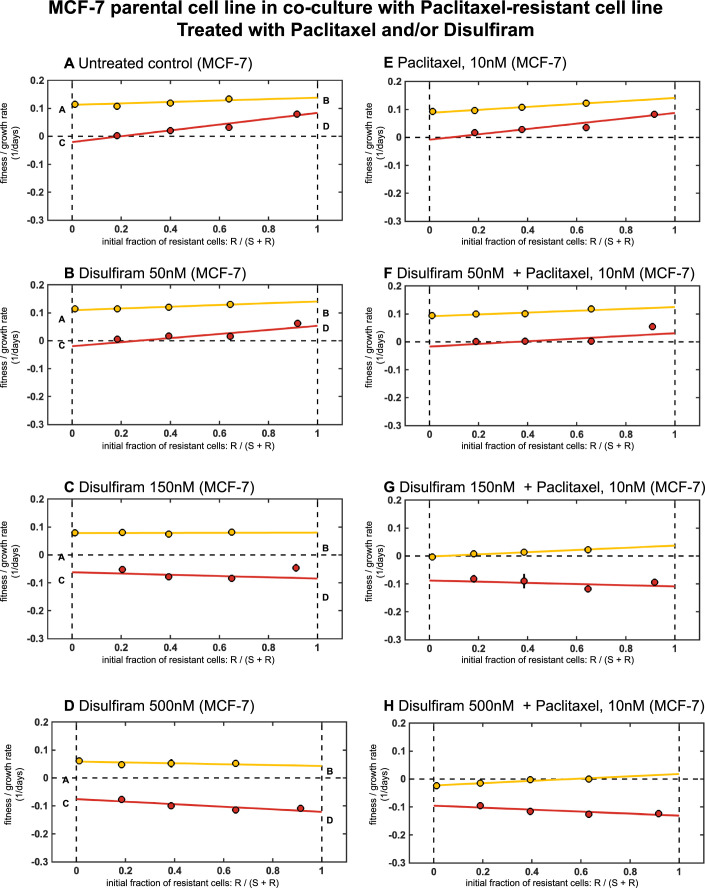
Growth rates (mean and standard deviation shown for three replicates at each ratio) for the corresponding coculture experiments in Fig. EV3 determine the cell type’s fitness as a function of resistant fraction. A linear fitness function is fit with parameters A, B, C, D denoted (See Methods). Shown for MCF-7 parental cell line in coculture with Paclitaxel-resistant cell line treated with Paclitaxel and/or Disulfiram. (**A**) Untreated control (MCF7). (**B**) Disulfiram, 50 nM (MCF7). (**C**) Disulfiram, 150 nM (MCF7). (**D**) Disulfiram, 500 nM (MCF7). (**E**) Paclitaxel, 10 nM (MCF7). (**F**) Disulfiram, 50 nM + Paclitaxel, 10 nM (MCF7). (**G**) Disulfiram, 150 nM + Paclitaxel, 10 nM (MCF7). (**H**) Disulfiram, 500 nM + Paclitaxel, 10 nM (MCF7).

**Figure EV7:**
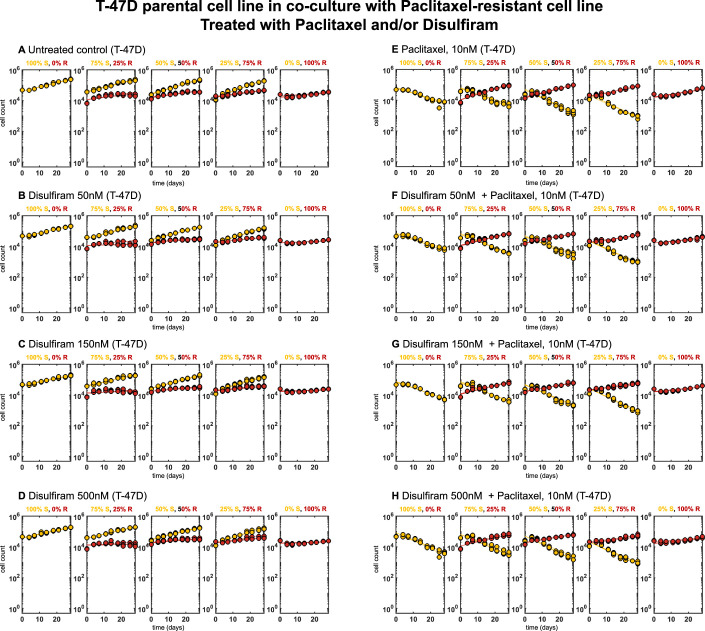
Evolutionary game assay: Cells are seeded with a ratio of resistant to naive of 0%R, 25%R, 50%R, 75%R, and 100%R across all treatment conditions. The best fit exponential growth rate is measured for each replicate (*n* = 3), repeated for each ratio. (**A**) Untreated control (T47D). (**B**) Disulfiram, 50 nM (T47D). (**C**) Disulfiram, 150 nM (T47D). (**D**) Disulfiram, 500 nM (T47D). (**E**) Paclitaxel, 10 nM (T47D). (**F**) Disulfiram, 50 nM + Paclitaxel, 10 nM (T47D). (**G**) Disulfiram, 150 nM + Paclitaxel, 10 nM (T47D). (**H**) Disulfiram, 500 nM + Paclitaxel, 10 nM (T47D).

**Figure EV8:**
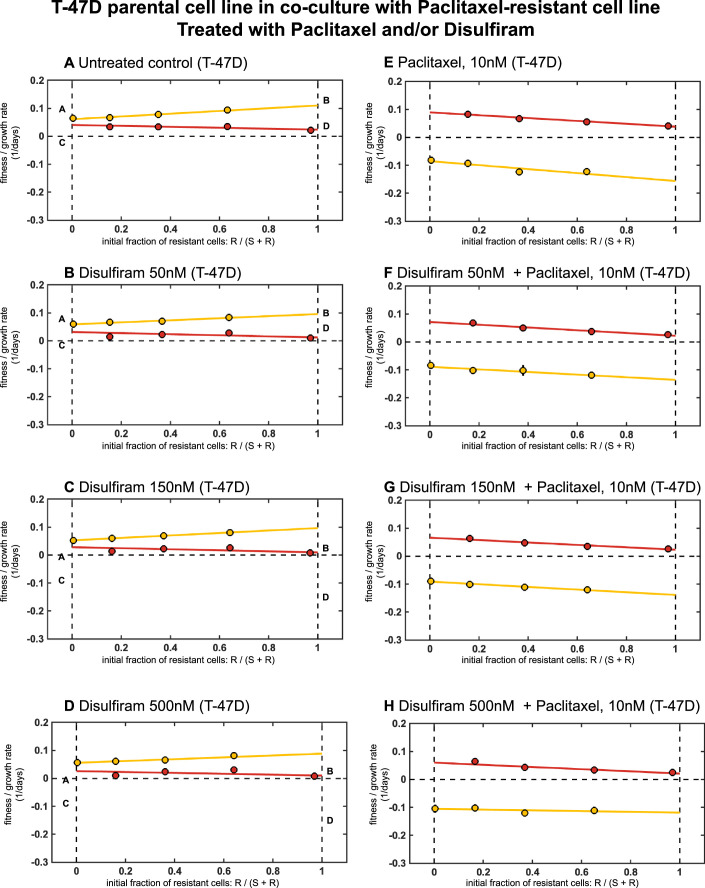
Growth rates (mean and standard deviation shown for three replicates at each ratio) for the corresponding coculture experiments in Fig. EV3 determine the cell type’s fitness as a function of resistant fraction. A linear fitness function is fit with parameters A, B, C, D denoted (See Methods). Shown for T-47D parental cell line in coculture with Paclitaxel-resistant cell line treated with Paclitaxel and/or Disulfiram. (**A**) Untreated control (T47D). (**B**) Disulfiram, 50 nM (T47D). (**C**) Disulfiram, 150 nM (T47D). (**D**) Disulfiram, 500 nM (T47D). (**E**) Paclitaxel, 10 nM (T47D). (**F**) Disulfiram, 50 nM + Paclitaxel, 10 nM (T47D). (**G**) Disulfiram, 150 nM + Paclitaxel, 10 nM (T47D). (**H**) Disulfiram, 500 nM + Paclitaxel, 10 nM (T47D).

Figure 4 quantifies ${\Delta }_{S}$ and ${\Delta }_{R}$ for a range of mono and combination therapy options for MCF-7 with doxorubicin-resistance (Figs. 4A and EV3A–H, EV4A–H), paclitaxel-resistance (Figs. 4B and EV5A–H, EV6A–H) and T-47D with paclitaxel-resistance (Fig. 4C). T-47D doxorubicin-resistant cells exhibited inconsistent proliferation rates, perhaps as a cost of acquired resistance, which presented challenges in growth and measurements for long-term organoid experiments. MCF-7 sensitive relative fitness (normalized growth rate) is always positive, while resistant relative fitness (normalized growth rate) is always negative $({\Delta }_{S} > 0,{\,\Delta }_{R} < 0)$. Disulfiram can be classified as a chemo-sensitizer, due to an increase in sensitivity with increasing disulfiram dose for both monotherapy and combination with chemo (Fig. 4A,B, left panel). This result is summarized in the middle panels of Fig. 4A,B, showing the four possible scenarios for selection dynamics: selection for sensitive (yellow quadrant), co-existence (orange), bistability (blue), or selection for resistance. In MCF7 lines, selection for sensitive cells is always observed under all treatment conditions in coculture, but introduction of disulfiram further shifts and strengthens the selection dynamics in favor of sensitive populations (Fig. 4A,B, middle panel). Selection for resistance is seen in the T47D line with chemotherapy in monotherapy or combination (Figs. 4C and EV7A–H, EV8A–H).

Next, we introduce a metric for the overall tumor fitness, ${\phi }^{* }$, defined as the long-term growth rate once the selection dynamics reach equilibrium (see Methods). Despite defining the selection dynamics, the overall spheroids may be growing (positive tumor fitness, ${\phi }^{* } > 0$) or decaying (negative tumor fitness, ${\phi }^{* } < 0$). For example, in all three cell lines, the untreated conditions select for sensitive lines, but maintain positive tumor fitness. In contrast, combination treatment of chemo with maximum disulfiram concentration leads to the lowest (negative) tumor fitness, indicating a negative tumor growth. Note: en route to tumor regression, the model predicts MCF-7 lines become more sensitive (${\Delta }_{S} > 0,\,{\Delta }_{R} < 0$) while T-47D lines become more resistant (${\Delta }_{S} < 0,\,{\Delta }_{R} > 0$).

### Evolutionary game assay validation

Thus far, we have considered only continuous therapeutic regimens: continuous dosing of a single therapy (chemotherapy or disulfiram alone) or continuous dosing of combination chemotherapy with disulfiram. As the mathematical model underlying the EGA technique is parameterized to continuous dosing data, it remains an open question if it’s useful to identify promising sequential therapeutic options (e.g., chemotherapy followed by disulfiram). Next, we consider a range of candidate sequential treatment regimens: alternating chemotherapy and disulfiram with fast (every week) or slow (every 2 weeks) drug switching. We do not consider any sequential schedule with disulfiram first, as previous figures indicate disulfiram performs best after chemo-resistance is selected for. Note: results from Fig. 4 intuitively suggest that sequential schedules are unlikely to outperform combination treatments, because tumor fitness (growth rate) is maximally negative under combination treatment, but positive under monotherapy of either chemo or disulfiram (Fig. 4A–C; right panels).

**Figure 5:**
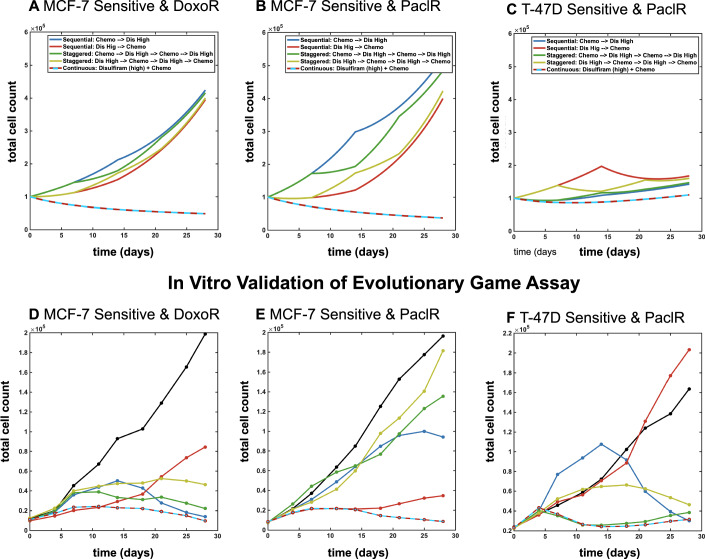
Model predictions for continuous and alternating treatments. Mathematical model simulations, parameterized in Fig. 4, shown for MCF-7 (**A**, **B**, **D**, **E**) and T47D (**C**, **F**) cell lines, respectively. Top panels (**A**, **B**, **C**): in silico simulations show spheroid cell count over time for continuous mono- and combination therapy and alternating/sequential therapy for fast and slow switching. Bottom panels (**D**, **E**, **F**): in vitro validation of model predictions for spheroid cell count over time for all therapy regimens. Treatments include chemotherapy concentration of 25 nM doxorubicin and 500 nM disulfiram. Source data are available online for this figure.

This intuition is confirmed in mathematical model simulations in Fig. 5, where combination treatment outperforms these sequential therapeutic regimens across parameterizations for each cell line (Fig. 5A–C; top panel). To explore the validity of these in silico predictions, our 3D cell culture model was confronted with the different drug treatments. Fluorescently labeled sensitive and chemo-resistant cells were once again plated at various population frequencies previously outlined in Fig. 3. Co-cultured spheroids were then treated with 25 nM of doxorubicin and/or 500 nM of disulfiram in continuous combination therapy or alternating/sequential therapeutic strategies. Each sequential regimen explored the possibility of leading treatment with either disulfiram or chemotherapy in an effort to investigate early selection trends of resistant and sensitive populations dependent on initial drug pressure.

Theoretical results are experimentally validated in Fig. 5 (Fig. 5D–F; bottom panel). Interestingly, in MCF7 lines, leading with disulfiram in alternating/sequential strategies produces more effective control of overall cell count compared with initiating treatment with chemotherapy. However, spheroid cell count is most negatively impacted under combination treatment out of all regimens. Therefore, the in vitro experiments confirm that combination therapy outperforms all sequential therapy regimens and is ideal across cell lines and initial sensitive-resistant fractions (shown left-to-right in bar groups).

## Discussion

A promising strategy for combating treatment resistance is to identify a candidate drug that can be classified as an evolutionary double-bind, whereby resistance to a first-line therapy confers sensitivity to a second-line treatment. In this work, we have identified disulfiram as the first reported example of an evolutionary double-bind with chemotherapy in ER+ breast cancer. To confirm the evolutionary double-bind, we developed an integrated mathematical-experimental framework to screen for promising candidate drugs.

Here, we developed resistant cell lineages over the course of long-term exposure to either paclitaxel or doxorubicin, and fluorescently labeled each cell line to track growth and treatment response over time. An extensive drug screen was performed using a range of candidate drugs either currently approved for use in breast cancer, exhibiting potential off-label use in oncological settings, or that inhibit pathways linked to resistance. From a panel of over 30 drug candidates, disulfiram was a leading drug found to be the most effective at inhibiting cell survival in all three chemo-resistant cell lineages compared to their respective chemo-sensitive cell lines. Cells resistant to paclitaxel harbored cross resistance to doxorubicin, but both resistant lines also harbored mutual sensitivity to disulfiram. This suggests a commonality between resistance mechanisms across these different chemotherapies. This commonality leads to a shared mechanism that disulfiram can exploit.

There are several important novelties implemented in this drug screening methodology. First, it is uncommon to quantify drug synergy in both parental and evolved-resistance cell lines to note changes in collateral sensitivity after the onset of resistance. Secondly, it is an open question how drug-drug synergy relates to cell–cell interactions between parental and evolved-resistance lines. Finally, it is unclear how to best implement drug scheduling and timing for candidate evolutionary double-bind treatments. The integrated mathematical-experimental framework outlined above addresses all these key questions.

### Disulfiram as an evolutionary double-bind with chemotherapy

From our comprehensive drug screen, we identified disulfiram as a candidate drug to engage in an evolutionary double-bind, capitalizing on secondary vulnerabilities arising in chemo-resistant cells. We quantified synergy by performing dose-response analyses on both chemo-sensitive and resistant lines treated with disulfiram in combination with either paclitaxel or doxorubicin. The quantification of drug synergy (Table 1) illustrates a moderate degree of heterogeneity across cell lines. Interestingly, synergistic potency is rarely seen in naive cell lines, but does appear in some resistant lines (e.g., MCF-7 DoxoR and T-47D DoxoR lines). Synergistic efficacy is more common and seen in both naive and resistant lines. Another promising result is that antagonism is rarely observed during disulfiram treatment for these lines. Interestingly, the presence of chemotherapy stabilizes the efficacy bifurcation of disulfiram observed when given alone. It is also important to note that even drugs without synergy may benefit from combination therapy due to an additive effect (Hwangbo et al, 2023; Schmidt et al, 2023; Palmer et al, 2022).

The relationship between drug synergy in resistant lines and corresponding cell–cell interactions between parental and resistant lines is under-explored. To quantify cell–cell interactions, we performed the evolutionary game assay (EGA) to measure the frequency-dependent growth of parental and resistant cells when co-cultured and treated with disulfiram, chemotherapy, and combination therapy. The EGA analysis confirms that disulfiram inhibits the fitness (growth rate) of chemo-resistant lines, causing re-sensitization to chemotherapy (Fig. 4). This is true across both doxorubicin and paclitaxel. Combination chemo-disulfiram results in the lowest total tumor fitness, across all cell lines. These results suggest that the two-drug combination is an effective combination with one drug to target sensitive cells (chemotherapy) and a second to target resistant cells (disulfiram). EGA results also suggest that the combination treatment would outperform any sequential treatment paradigm. This result was validated in vitro, confirming the optimality of combination treatment (Fig. 5).

Disulfiram is an FDA-approved anti-alcoholism drug reported to also have anti-tumor activity in several pre-clinical studies and clinical trials. Disulfiram has shown strong tumor-selective toxicity and inhibition of cancer cell growth (Iljin et al, 2009). Repurposing drugs approved for other indications and expanding them for use in oncological settings brings many advantages. Drug repurposing decreases significant time and costs traditionally invested in research and development. It also reduces the drug development time frame by providing valuable safety data demonstrated in previous clinical trials. Several clinical trials have investigated disulfiram as a combination therapy with chemotherapy in solid tumors. For example, there are active trials in metastatic pancreatic cancer (NCT02671890) and treatment-refractory sarcoma (NCT05210374), while a completed trial in recurrent glioblastoma (NCT02678975) showed no statistical significant difference in 6-month survival(Werlenius et al, 2023). For these reasons, disulfiram presents as a promising drug in refractory metastatic breast cancer, prompting greater investigation for combination treatment with chemotherapy in our current study.

While specific molecular mechanisms conferring vulnerability to disulfiram in resistant cells were not explored in the current study, future aims will center around elucidating mechanistic targets related to disulfiram in metastatic breast cancer. Disulfiram is known to suppress angiogenesis (Marikovsky et al, 2002; Li et al, 2015), constrain tumor metastasis (Duan et al, 2014; Bu et al, 2019), and modulate the immune microenvironment (Zhao et al, 2018; Hu et al, 2020). Previous studies have also shown disulfiram to be an inhibitor of PI3K signaling, often dysregulated in breast cancer, leading to suppression of cell proliferation and survival (Zhang et al, 2010). MDR drug efflux pump expression differences are often found in chemo-resistant cells compared to sensitive cells, and recent studies have also presented evidence that disulfiram may combat malignancy by inhibiting the ABC drug transport proteins involved in drug resistance (Loo et al, 2004; Loo and Clarke, 2000; Choi and Yu, 2014). Disulfiram has been shown to inhibit NF-kB (involved in epithelial-mesenchymal transition) and the self-renewal of breast cancer stem cells (Han et al, 2015). Disulfiram metabolites have also been shown to induce p53, a key player in maintaining the balance between self-renewal and differentiation, ultimately leading to apoptosis and cell death (Kanellis et al, 2023). Therefore, while the exact acquired resistance trait targeted by disulfiram in these studies is not yet known, molecular aberrations in these pathways and processes may render these cells vulnerable to disulfiram.

To strengthen disulfiram as a candidate drug for potential application in clinical oncology settings, further in vitro as well as in vivo testing of combination therapy with disulfiram would be necessary to determine the optimal dosing, timing strategy, and toxicity concerns for future clinical trials. Combination disulfiram with chemotherapy resulted in the best drug response. Disulfiram alone was associated with a non-monotonic dose response (worse response for higher doses), which was not observed when combined with chemotherapy (Fig. 2).

Additional dosing schemes should also consider combination treatment of disulfiram along with alternative agents, given it is unlikely disulfiram would be clinically administered as a monotherapy. For example, previous studies have shown disulfiram-copper complexes can induce apoptosis in prostate cancer (Thoma, 2014). Through integration of experimental and mathematical models, this work highlights the importance of identifying targetable drug sensitivities in chemo-resistant settings and the potential to predict and avoid selection of resistant populations under therapy. Future investigations will explore further applications of the evolutionary game assay as it may be expanded to include other resistant cell lines, treatment strategies, and dosing concentrations. Elucidating the underlying molecular mechanisms involved in chemo-resistance may also allow for a deeper understanding of the mechanistic drivers of the observed evolutionary double-bind and the potential for clinical applications of disulfiram in combination therapy.

## Methods

**Table:** Reagents and tools table

Reagent/resource	Reference or source	Identifier or catalog number
**Experimental models**		
T47D cells (*H. sapiens*)	ATCC	HTB-133
MCF-7 cells (*H. sapiens*)	ATCC	HTB-22
T47D_V2	This study	
MCF-7_V2	This study	
T47D evolved paclitaxel-resistance/T47D_C2	This study	
T47D evolved doxorubicin-resistance/T47D_C2	This study	
MCF-7 evolved paclitaxel-resistance/MCF-7_C2	This study	
MCF-7 evolved doxorubicin-resistance/MCF-7_C2	This study	
**Recombinant DNA**		
LeGO-V2	Addgene, Weber et al, 2008	Plasmids #27340
LeGO-C2	Addgene, Weber et al, 2008	Plasmids #27339
**Antibodies**		
**Oligonucleotides and other sequence-based reagents**		
**Chemicals, enzymes and other reagents**		
DMEM, high glucose, pyruvate	Gibco	11995065
RPMI 1640 Medium	Gibco	11875093
Fetal bovine serum (FBS)	Millipore Sigma	12306C
Mycoplasma-negative using the Mycoalert PLUS Mycoplasma detection kit	Lonza	LT07-703
CellTiter-Glo® Luminescent Cell Viability Assay Kit	Promega	G7573
Dimethyl Sulfoxide, Fisher BioReagents™	Fisher Scientific	BP231-100
Antibiotic–Antimycotic (100X)	Gibco	15240062
Gefitinib	Selleckchem	S1025
Erlotinib	Selleckchem	S7786
Sorafenib	Selleckchem	S7379
Sunitinib	Selleckchem	S7781
Dasatinib	Selleckchem	S1021
Bosutinib	Selleckchem	S1014
C188-9	Selleckchem	S8605
BP-1-102	Selleckchem	S7769
GDC-0941	Selleckchem	S1065
BYL719	Selleckchem	S2814
Taselisib (GDC 0032)	Selleckchem	S7103
Bimiralisib (PQR309)	Selleckchem	S8738
Everolimus	Selleckchem	S1120
MLN0128	Selleckchem	S2811
Trametinib	Selleckchem	S2673
Binimetinib (MEK162)	Selleckchem	S7007
Belinostat	Selleckchem	S1085
Entinostat	Selleckchem	S1053
Ribociclib	Selleckchem	S7440
Abemaciclib	Selleckchem	S5716
Doxorubicin	Selleckchem	S1208
Paclitaxel	Selleckchem	S1150
Carboplatin	Selleckchem	S1215
Gemcitabine	Selleckchem	S1714
5-fluoro-uracil	Selleckchem	S1209
Momelotinib (CYT387)	Selleckchem	CYT387
Linsitinib	Selleckchem	S1091
Olaparib	Selleckchem	S1060
Veliparib (ABT-888)	Selleckchem	S1004
Azacitidine	Selleckchem	S1782
Adavosertib (MK-1775)	Selleckchem	S1525
A673	Tocris	6934
Disulfiram	Selleckchem	S1680
AZD4547	Selleckchem	S2801
ICG-001	Selleckchem	S2662
OTX015	Selleckchem	S7360
Ipatasertib (GDC-0068)	Selleckchem	S2808
**Software**		
MatLab	Mathworks.com	24.2.0.2773142 (R2024b)
**Other Materials**		
Tubes and Flat Caps, strips of 8	Thermo Scientific	AB1182
Corning™ 384-Well, Cell Culture-Treated, Flat-Bottom, Low Flange Microplate	Corning	3764
Corning™ 96-Well, Ultra-Low Binding, U-Shaped-Bottom Microplate	Corning	4520

### Cell lines and reagents

The previously authenticated estrogen-receptor-positive (ER+) T-47D (ATCC, # HTB-133) and MCF-7 breast cancer cell lines (ATCC, # HTB-22) were maintained in RPMI + 10% FBS + 1% antibiotic–antimycotic or DMEM + 10% FBS + 1% antibiotic–antimycotic solution, respectively. The chemo-resistant cell line creation (paclitaxel-resistant T-47D; doxorubicin-resistant T-47D; paclitaxel-resistant MCF-7; doxorubicin-resistant MCF-7) was performed by culturing cells in regular maintenance media with weekly 24-h exposure in chemotherapy-treated media, using doxorubicin (Selleck Chemicals, Cat. No: E2516) or paclitaxel (Selleck Chemicals, Cat. No: S1150), at different concentrations. Briefly, once a week and for a 24-h window, cells were cultured in 70 nM doxorubicin treated media (doxorubicin-resistant T-47D), 80 nM doxorubicin treated media (doxorubicin-resistant MCF-7), 30 nM paclitaxel (paclitaxel-resistant T-47D), or 80 nM paclitaxel (paclitaxel-resistant MCF-7). This was performed over a 6–8 month course of time to develop resistance. Resistance against chemotherapy was detected by the alteration of the dose-response curve measured using CellTiter-Glo Chemiluminescent Kit (Promega Corporation, Cat. No.: G7573). Cell lines were confirmed to be mycoplasma-negative using the Mycoalert PLUS Mycoplasma detection kit (Lonza, Cat. No.: LT07-703).

### Lentiviral labeling of sensitive and resistant cells

Using lentiviruses incorporating distinct fluorescent proteins, we labeled T-47D and MCF-7 parental sensitive cells (Venus; LeGO-V2) and chemotherapy-resistant cells (mCherry; LeGO-C2), as earlier reported(Grolmusz et al, 2020). LeGO-V2 and LeGO-C2 vectors were provided by Boris Fehse (Addgene plasmids #27340 and #27339). Lentiviruses with fluorescent proteins were created using Lipofectamine 3000 reagent (Thermo Fisher Scientific) following the manufacturer’s protocol. T-47D and MCF-7 sensitive and resistant cell lines were transduced with lentivirus using reverse transduction. Briefly, 1 mL of polybrene-containing cell suspension of 200,000 cells were plated in a well of a six-well plate. Previously, 0.5 mL of viral aliquot had been dispensed in a plate. Following 48 h of incubation at 37 °C with 5% CO_2_, cells were washed and given fresh regular culture medium. To select for fluorescence-activated cells, fluorescently labeled cells were flow-sorted after further subculture of transduced cells to attain homogeneously labeled cell populations.

### Drug screening and dose response experiments with sensitive and resistant cell lines

T-47D and MCF-7 sensitive and doxorubicin or paclitaxel-resistant cells were used in a drug screen and treated with various therapies. Therapies used in the drug screen are summarized in Fig. 1A. About 2000 cells were plated in 384 flat-bottom well plates (Corning, Cat. No. 142761) on Day 0 and treated 24 h from plating (Day 1) for a duration of 72 h. After 72 h, collateral sensitivity against each drug was detected by measuring the viability of each line using CellTiter-Glo Chemoluminescent Kit (Promega Corporation, Cat. No.: G7573). Relative viability of each resistant cell under each condition was normalized to parental sensitive cells under the same condition and were subjected to log2-transformation. All 2D dose response experiments following the drug screen for mono- and combo-therapy with disulfiram and chemotherapy were plated, drugged, and analyzed in the same manner and performed in triplicates.

### Mono- and coculture 3D spheroid experiments

The 18–25-day experiments were initiated with fluorescently labeled sensitive and resistant cell lines in different compositions. For long-term T-47D and MCF-7 spheroid experiments, 5000 cells were plated in different proportions (100% sensitive, 75/25 sensitive-resistant, 50/50 sensitive-resistant, and 25/75 sensitive-resistant) in a 96-well round-bottom ultra-low attachment spheroid microplate (Corning, Cat. No.: 4520). Twenty-four hours later, spheroids were washed, and fresh medium including treatment drugs was applied (day 0). Spheroids were treated for a total of 18–25 days, with imaging and media change performed every 4th and 7th day of the week. Spheroids were treated with either doxorubicin (Selleck Chemicals, Cat. No: E2516) or paclitaxel (Selleck Chemicals, Cat. No: S1150) at specified doses and various strategies (either continuous, staggered, layered, or sequential) as described in the results and Figs. 3–5. Imaging was performed using Cytation 5 imager (Biotek Instruments), recording signal intensity from brightfield, YFP (for Venus fluorescence), and Texas Red (for mCherry fluorescence) channels. Raw data processing and image analysis were performed using Gen5 3.05 and 3.10 software (Biotek Instruments). Briefly, the stitching of 2 × 2 montage images and Z-projection of six layers using focus stacking was performed on raw images, followed by spheroid area analysis. To quantify growth under these conditions, we measured fluorescence intensity or relative sensitivity and resistant populations and the growth of spheroid area over the total time of the experiment. For cell count calculations, a standard curve was created by measuring the fluorescence and area of spheroids, 24 h after plating at different cell numbers (a range of 500 cells to 250,000 cells) and at different proportions (100% sensitive, 80/20 resistant-sensitive, 60/40 resistant-sensitive, 40/60 resistant-sensitive, 20/80 resistant-sensitive, 100% resistant). Further details regarding 3D cell count quantification can be found under “Cell number quantification“ in Methods (below). All coculture experiments were performed in triplicates.

### Cell number quantification

The following method was used to estimate the number of cells of each type based on fluorescent imaging data. The number of cells, ${{{{\rm{N}}}}}_{i}$ of type *i* ($i=[S,R]$), is estimated by fluorescent intensity, ${{{{\rm{F}}}}}_{i}$, according to the following equation:

$${log }_{10}\left({N}_{i}\right)=a{log }_{10}\left({F}_{i}\right)+b$$

where *a* and *b* are constant values that differ for each cell line (MCF-7, T-47D) and for each evolved resistance line (parental, paclitaxel, doxorubicin). To correct for possible differences in per-cell fluorescence, we regressed the fluorescence of pure cultures against the known numbers of S and R cells (Fig. EV2), where the seeded cell number (x-axis) should match the predicted cell number (y-axis) across all initial sizes and sensitive-to-resistant ratios (circle color).

### Dose response

Treatment sensitivity is often modeled using a Hill equation. The Hill function can be derived by considering a weighted average of unaffected cells (${E}_{0}$), affected cells (${E}_{1}$), resulting in the equilibrium of a reversible transformation between these two populations, with corresponding dose-dependent rates of action (Meyer et al, 2019a). The viability of a population of cells as a function of dose, x, can be written:

1 $$H\left(x\right)=\frac{{E}_{1}-{E}_{0}}{1+{\left(\frac{C}{x}\right)}^{n}}+{E}_{0}$$

The function in Eq. 1 can be extended to consider two drugs with synergistic or antagonistic effects. Drug synergy or antagonism can be estimated by fitting a dose response surface, $H\left({x}_{1},{x}_{2}\right)$, to a checkerboard dose response assay(Meyer et al, 2020; Wooten et al, 2021; Meyer et al, 2019b). representing combinations of drugs 1 and 2 at various concentrations, ${x}_{1}$ and ${x}_{2}$, respectively. For drugs that obey detailed balance, the two-dimensional dose response is given by:

2 $$H\left({{x}_{1},x}_{2}\right)=\frac{{{C}_{1}}^{{h}_{1}}{{C}_{2}}^{{h}_{2}}{E}_{0}+{{x}_{1}}^{{h}_{1}}{{C}_{2}}^{{h}_{2}}{E}_{1}+{{C}_{1}}^{{h}_{1}}{{x}_{2}}^{{h}_{2}}{E}_{2}+{({a}_{21}{x}_{1})}^{{h}_{1}}{{x}_{2}}^{{h}_{2}}{E}_{3}}{{{C}_{1}}^{{h}_{1}}{{C}_{2}}^{{h}_{2}}+{{x}_{1}}^{{h}_{1}}{{C}_{2}}^{{h}_{2}}+{{C}_{1}}^{{h}_{1}}{{x}_{2}}^{{h}_{2}}+{({a}_{21}{x}_{1})}^{{h}_{1}}{{x}_{2}}^{{h}_{2}}}$$

### Evolutionary game assay

The mathematical modeling follows the evolutionary game assay outlined in ref. (Kaznatcheev et al, 2019a), which we restate here briefly. Growth dynamics can be classified as having frequency-dependence if the fitness function changes with the seeded ratio (frequency) of resistant cells. Fitness is a linear function of the monoculture growth rates for sensitive and resistant lines. The model has four parameters that comprise the payoff matrix, **P**, providing a complete characterization of the fitness function of two competing cell lines. The evolutionary game assay is given by the following set of ordinary differential equations:

3 $${{{\bf{P}}}}=\left(\begin{array}{cc}A & B\\ C & D\end{array}\right)\Rightarrow \left\{\begin{array}{ccc}\frac{{dS}}{{dt}} & = & S\left(A\frac{S}{S+R}+B\frac{R}{S+R}\right)\\ \frac{{dR}}{{dt}} & = & R\left(C\frac{S}{S+R}+D\frac{R}{S+R}\right)\end{array}\right.$$

The two equations can be thought of as exponential growth with an instantaneous growth rate that is a linear function of the proportion of each cell type. For example, the equations above can be normalized to track the dynamics of the proportion of sensitive cells, which reduces to the replicator equation:

4 $$\frac{d\hat{S}}{{dt}}=\hat{S}(1-\hat{S})[(B-D)(1-\hat{S})+(C-A)\hat{S}]$$

Thus, we define two important quantities that determine the selection dynamics in this model system: ${\Delta }_{S}$ and ${\Delta }_{R}$.

$${\Delta }_{S}=B-D$$

and

$${\Delta }_{R}=C-A$$

These two quantities represent the invasion fitness for each cell type when the proportion of that cell type is small. For example, if ${\Delta }_{R} > 0$, resistant cells are expected to invade a population of sensitive cells. Whether that invasion continues to full dominance (resistant cells fully take over) depends on the value of ${\Delta }_{S}$. Finally, we denote the total tumor growth rate, ${dS}/{dt}+{dR}/{dt}$, (tumor fitness) at long-term equilibrium (i.e., $d\hat{S}/{dt}=0$), as ${{{{\rm{\phi }}}}}^{* }$.

For all experiments, no blinding procedures were necessary for this study.

## Supplementary information

Peer Review File

Source data Fig. 1

Source data Fig. 2

Source data Fig. 3

Source data Fig. 4

Source data Fig. 5

Expanded View Figures

## Data Availability

The datasets and computer code produced in this study are available in the following database: https://github.com/MathOnco/ER-breast-cancer-evolutionary-double-bind. The source data of this paper are collected in the following database record: biostudies:S-SCDT-10_1038-S44320-026-00191-z.
