# Supplementary material for: A novel combination therapy for ER+ breast cancer suppresses drug resistance via an evolutionary double-bind
Source: Mol Syst Biol. 2026 Mar 26;22(7):1070–96. doi: 10.1038/s44320-026-00191-z (PMC13328575; doi:10.1038/s44320-026-00191-z)
Supplement: Supplementary file 7 — Expanded View Figures [file 44320_2026_191_MOESM7_ESM.pdf]

Expanded View Figures

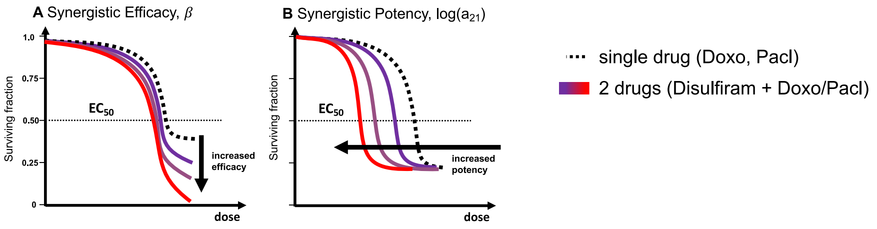

Figure EV1. Schematic representation of MUSYC parameters describing synergistic potency and synergistic efficacy.

(**A**) Synergistic efficacy, β, leads to a decrease in surviving fraction at high doses when the second drug is delivered in combination. (**B**) Synergistic potency, $\log(a_{21})$, leads to a decrease in the EC_{50} concentration when the second drug is delivered in combination.

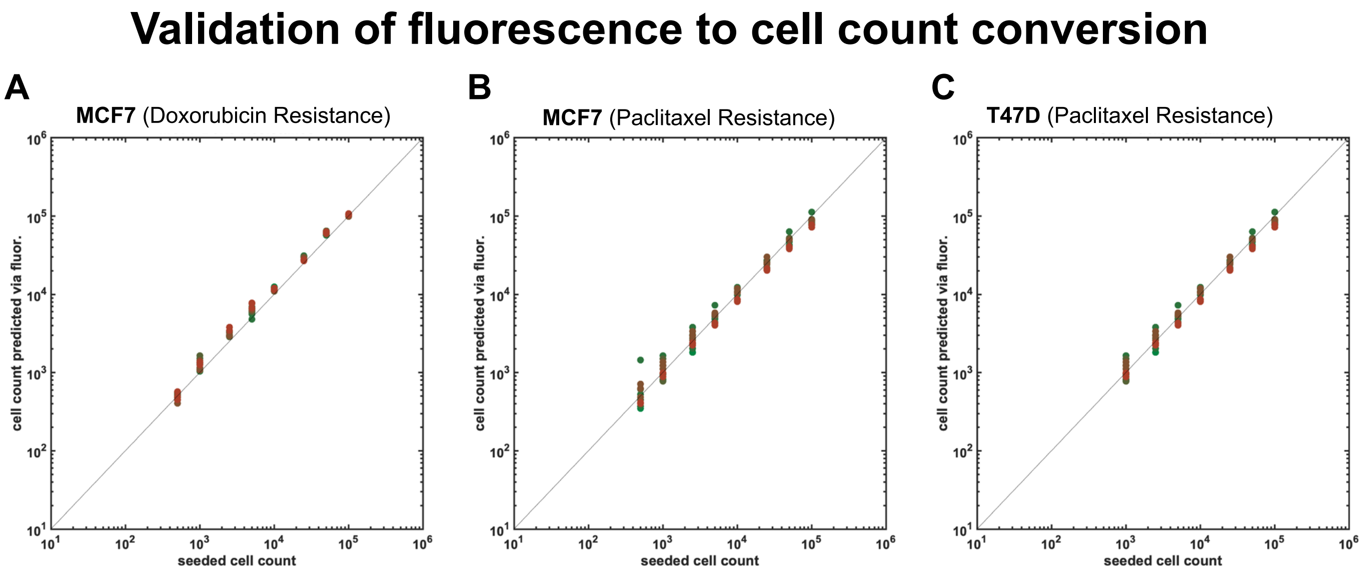

Figure EV2. Validation of fluorescence intensity, F, to cell count, N, equation (see Methods).

(**A**) Fluorescence to cell count conversion for MCF7 (Doxorubicin resistance). (**B**) Fluorescence to cell count conversion for MCF7 (Paclitaxel-resistance). (**C**) Fluorescence to cell count conversion for T47D (Paclitaxel-resistance). Known values of cells seeded (x-axis) are compared to predicted cell counts based on measured fluorescence intensity (y-axis). The model provides accurate cell count predictions, falling on the unity line (black line) across all initial sensitive-to-resistant ratios (color).

 © The Author(s)

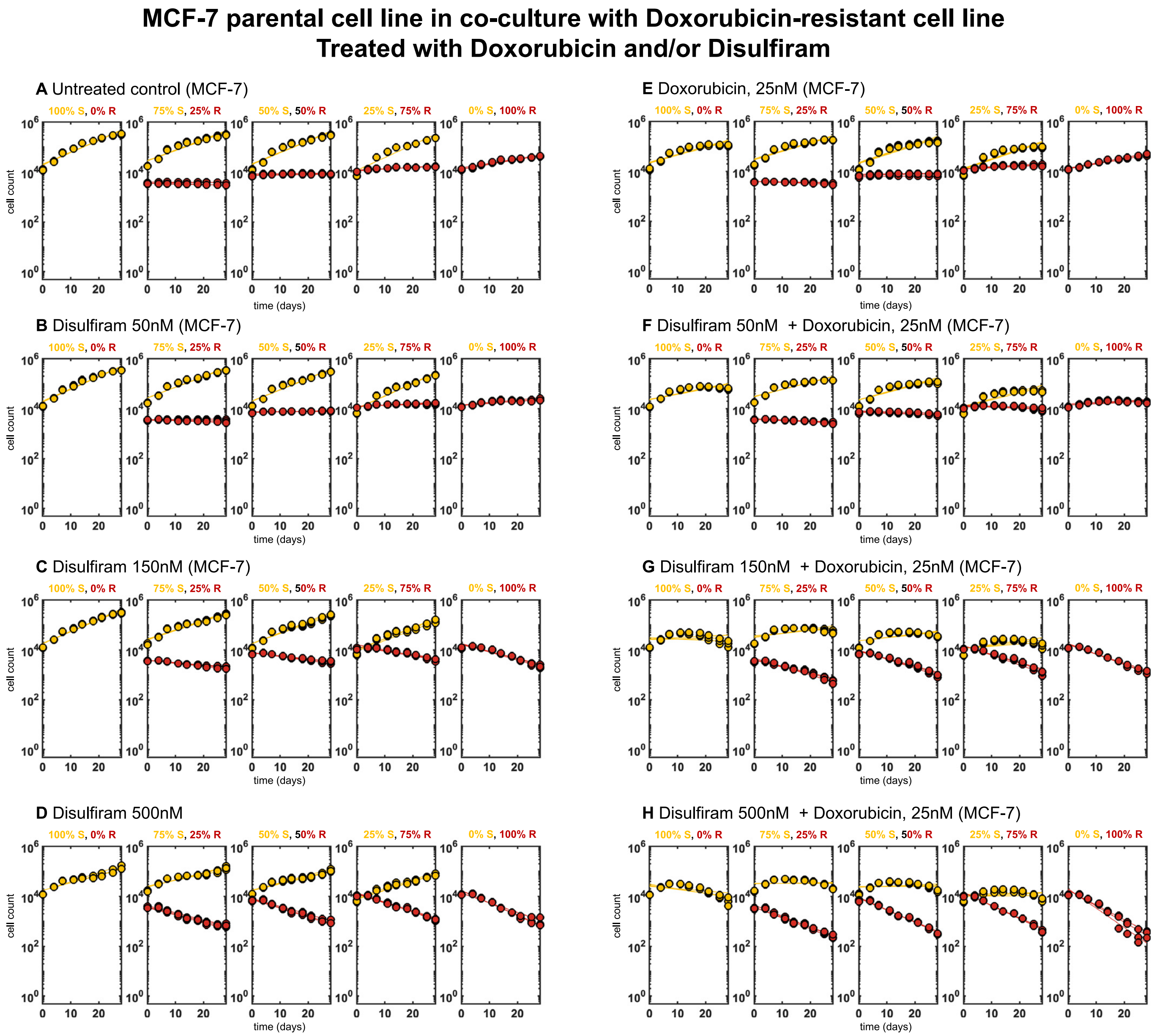

Figure EV3. Evolutionary game assay: Cells are seeded with a ratio of resistant to naive of 0%R, 25%R, 50%R, 75%R, and 100%R across all treatment conditions.

The best fit exponential growth rate is measured for each replicate ($n = 3$), repeated for each ratio. (**A**) Untreated control (MCF7). (**B**) Disulfiram, 50 nM (MCF7). (**C**) Disulfiram, 150 nM (MCF7). (**D**) Disulfiram, 500 nM (MCF7). (**E**) Doxorubicin, 25 nM (MCF7). (**F**) Disulfiram, 50 nM + Doxorubicin, 25 nM (MCF7). **G**) Disulfiram, 150 nM + Doxorubicin, 25 nM (MCF7). (**H**) Disulfiram, 500 nM + Doxorubicin, 25 nM (MCF7).

© The Author(s)

MCF-7 parental cell line in co-culture with Doxorubicin-resistant cell line Treated with Doxorubicin and/or Disulfiram

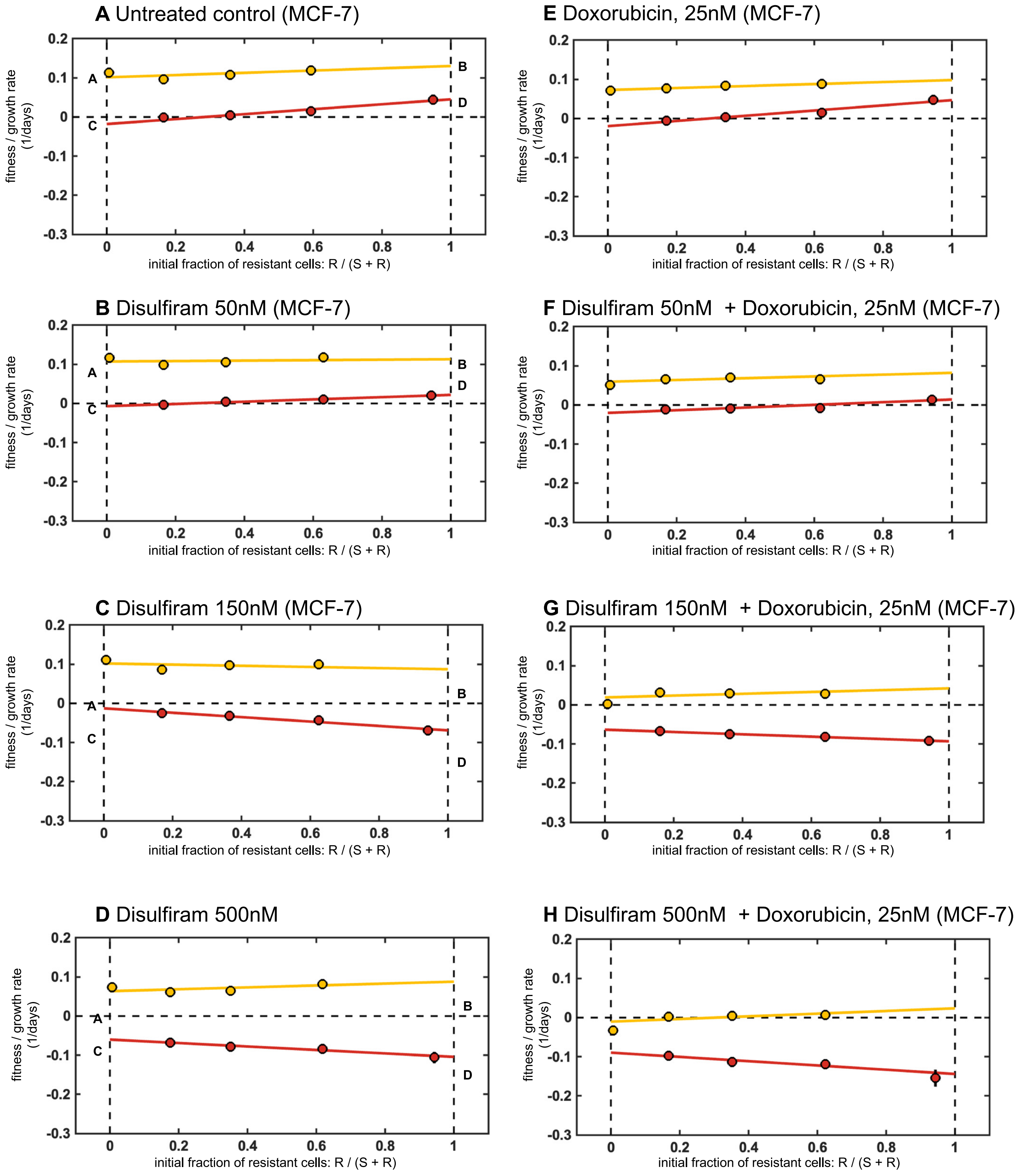

 © The Author(s)

Figure EV4. Growth rates (mean and standard deviation shown for 3 replicates at each ratio) for the corresponding coculture experiments in Fig. EV3 determine the cell type's fitness as a function of resistant fraction. A linear fitness function is fit with parameters A, B, C, D denoted (See Methods). Shown for MCF-7 parental cell line in coculture with Doxorubicin-resistant cell line, treated with Doxorubicin and/or Disulfiram as indicated. (**A**) Untreated control (MCF7). (**B**) Disulfiram, 50 nM (MCF7). (**C**) Disulfiram, 150 nM (MCF7). (**D**) Disulfiram, 500 nM (MCF7). (**E**) Doxorubicin, 25 nM (MCF7). (**F**) Disulfiram, 50 nM + Doxorubicin, 25 nM (MCF7). (**G**) Disulfiram, 150 nM + Doxorubicin, 25 nM (MCF7). (**H**) Disulfiram, 500 nM + Doxorubicin, 25 nM (MCF7).

© The Author(s)

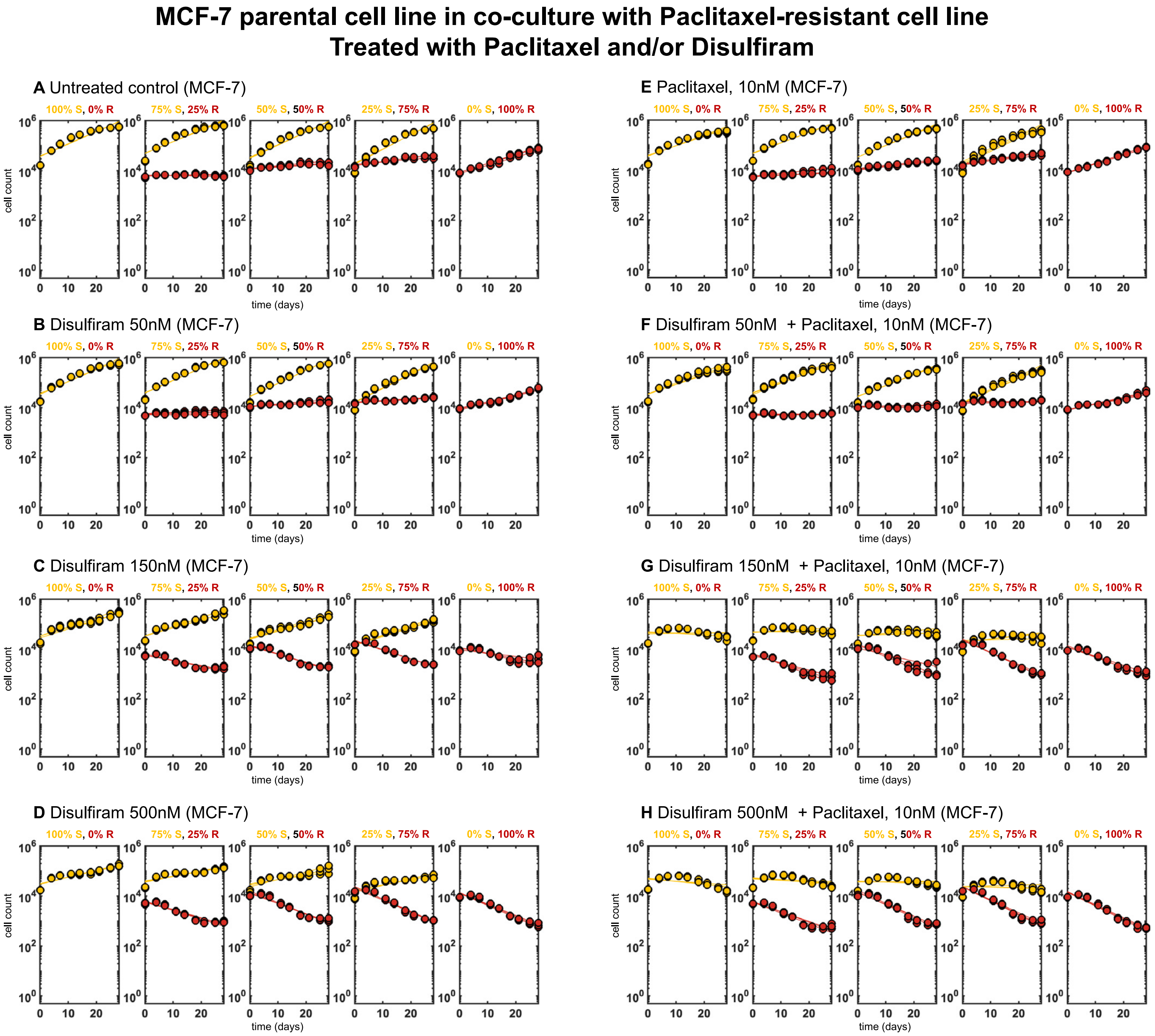

Figure EV5. Evolutionary game assay: Cells are seeded with a ratio of resistant to naive of 0%R, 25%R, 50%R, 75%R, and 100%R across all treatment conditions.

The best fit exponential growth rate is measured for each replicate ($n = 3$), repeated for each ratio. (**A**) Untreated control (MCF7). (**B**) Disulfiram, 50 nM (MCF7). (**C**) Disulfiram, 150 nM (MCF7). (**D**) Disulfiram, 500 nM (MCF7). (**E**) Paclitaxel, 10 nM (MCF7). (**F**) Disulfiram, 50 nM + Paclitaxel, 10 nM (MCF7). (**G**) Disulfiram, 150 nM + Paclitaxel, 10 nM (MCF7). (**H**) Disulfiram, 500 nM + Paclitaxel, 10 nM (MCF7).

 © The Author(s)

MCF-7 parental cell line in co-culture with Paclitaxel-resistant cell line Treated with Paclitaxel and/or Disulfiram

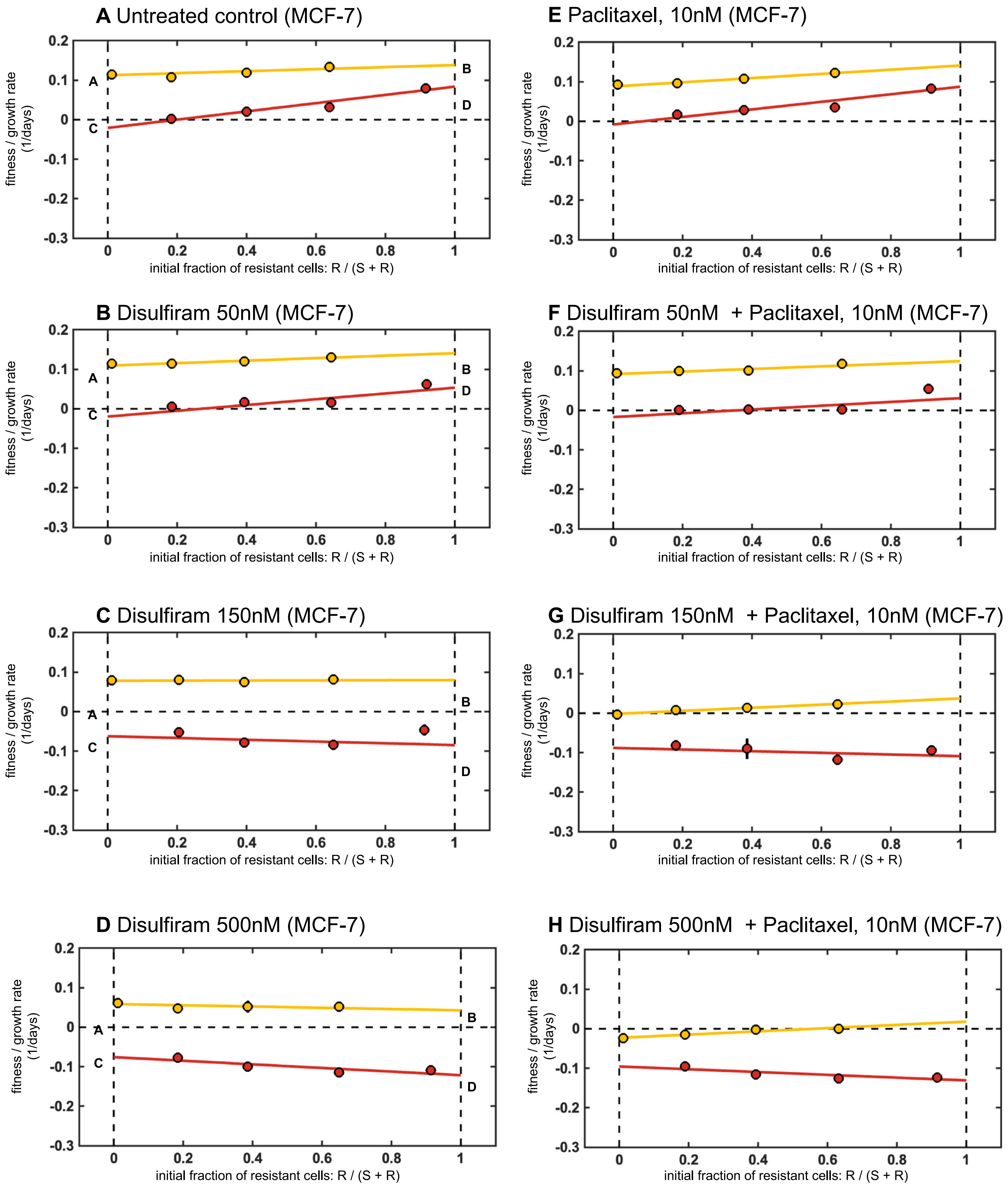

◀ **Figure EV6.** Growth rates (mean and standard deviation shown for three replicates at each ratio) for the corresponding coculture experiments in Fig. EV3 determine the cell type's fitness as a function of resistant fraction. A linear fitness function is fit with parameters A, B, C, D denoted (See Methods). Shown for MCF-7 parental cell line in coculture with Paclitaxel-resistant cell line treated with Paclitaxel and/or Disulfiram. (**A**) Untreated control (MCF7). (**B**) Disulfiram, 50 nM (MCF7). (**C**) Disulfiram, 150 nM (MCF7). (**D**) Disulfiram, 500 nM (MCF7). (**E**) Paclitaxel, 10 nM (MCF7). (**F**) Disulfiram, 50 nM + Paclitaxel, 10 nM (MCF7). (**G**) Disulfiram, 150 nM + Paclitaxel, 10 nM (MCF7). (**H**) Disulfiram, 500 nM + Paclitaxel, 10 nM (MCF7).

 © The Author(s)

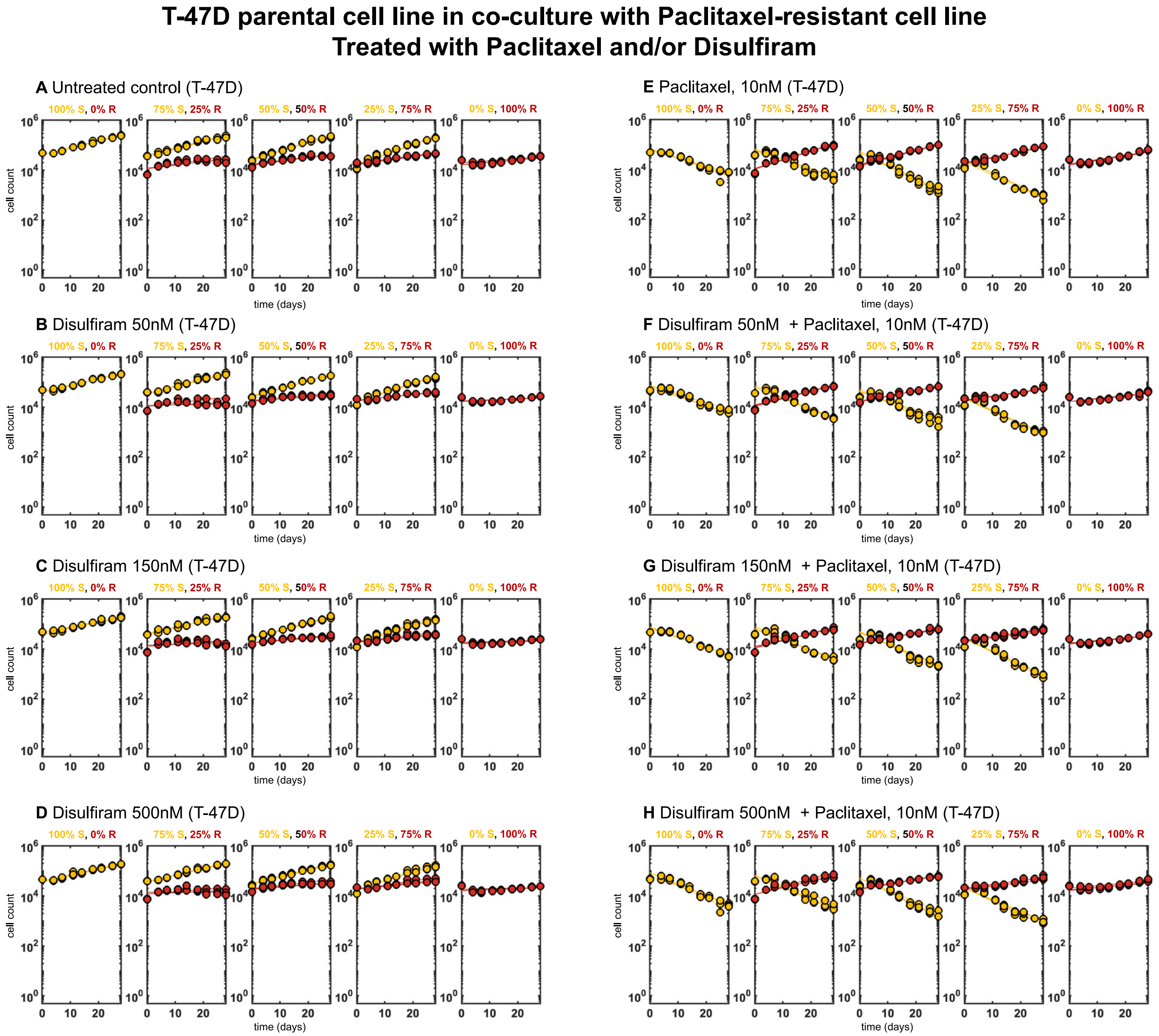

Figure EV7. Evolutionary game assay: Cells are seeded with a ratio of resistant to naive of 0%R, 25%R, 50%R, 75%R, and 100%R across all treatment conditions.

The best fit exponential growth rate is measured for each replicate ($n = 3$), repeated for each ratio. (**A**) Untreated control (T47D). (**B**) Disulfiram, 50 nM (T47D). (**C**) Disulfiram, 150 nM (T47D). (**D**) Disulfiram, 500 nM (T47D). (**E**) Paclitaxel, 10 nM (T47D). (**F**) Disulfiram, 50 nM + Paclitaxel, 10 nM (T47D). (**G**) Disulfiram, 150 nM + Paclitaxel, 10 nM (T47D). (**H**) Disulfiram, 500 nM + Paclitaxel, 10 nM (T47D).

T-47D parental cell line in co-culture with Paclitaxel-resistant cell line Treated with Paclitaxel and/or Disulfiram

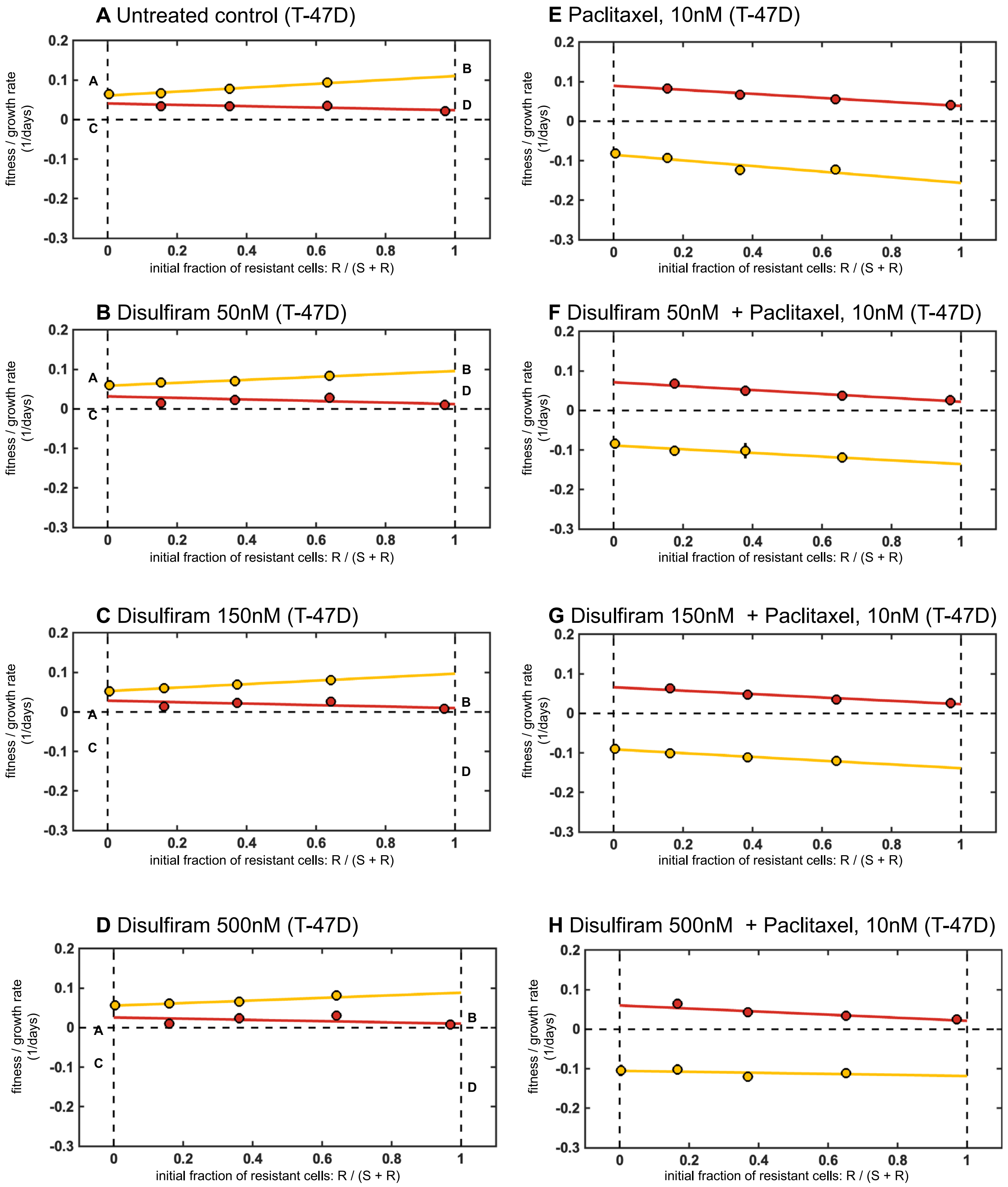

 © The Author(s)

Figure EV8. Growth rates (mean and standard deviation shown for three replicates at each ratio) for the corresponding coculture experiments in Fig. EV3 determine the cell type's fitness as a function of resistant fraction. A linear fitness function is fit with parameters A, B, C, D denoted (See Methods). Shown for T-47D parental cell line in coculture with Paclitaxel-resistant cell line treated with Paclitaxel and/or Disulfiram. (**A**) Untreated control (T47D). (**B**) Disulfiram, 50 nM (T47D). (**C**) Disulfiram, 150 nM (T47D). (**D**) Disulfiram, 500 nM (T47D). (**E**) Paclitaxel, 10 nM (T47D). (**F**) Disulfiram, 50 nM + Paclitaxel, 10 nM (T47D). (**G**) Disulfiram, 150 nM + Paclitaxel, 10 nM (T47D). (**H**) Disulfiram, 500 nM + Paclitaxel, 10 nM (T47D).
