# Supplementary material for: A novel combination therapy for ER+ breast cancer suppresses drug resistance via an evolutionary double-bind
Source: Mol Syst Biol. 2026 Mar 26;22(7):1070–96. doi: 10.1038/s44320-026-00191-z (PMC13328575; doi:10.1038/s44320-026-00191-z)
Supplement: Supplementary file 1 — Peer Review File [file 44320_2026_191_MOESM1_ESM.pdf]

molecular systems biology

A novel therapy for ER+ breast cancer suppresses drug resistance via an evolutionary double-bind

Rena Emond, Jeffrey West, Vince Grolmusz, Patrick Cosgrove, Aritro Nath, Alexander Anderson, and Andrea Bild

Corresponding author(s): Andrea Bild (abild@coh.org) , Alexander Anderson (alexander.anderson@moffitt.org), Andrea Bild (abild@coh.org)

Review Timeline:

Submission Date:	10th Jan 25
Editorial Decision:	15th Jan 25
Appeal Received:	31st Jan 25
Editorial Decision:	19th Mar 25
Revision Received:	5th Aug 25
Editorial Decision:	24th Oct 25
Revision Received:	3rd Dec 25
Accepted:	13th Jan 26

Editor: Poonam Bheda

Transaction Report:

(Note: With the exception of the correction of typographical or spelling errors that could be a source of ambiguity, letters and reports are not edited. Depending on transfer agreements, referee reports obtained elsewhere may or may not be included in this compilation. Referee reports are anonymous unless the Referee chooses to sign their reports.)

14th Jan 2025
RE: Manuscript MSB-2025-12854, A novel therapy for ER+ breast cancer suppresses drug resistance via an evolutionary double-bind

Dear Dr. Bild,

Thank you for having submitted a manuscript entitled "A novel therapy for ER+ breast cancer suppresses drug resistance via an evolutionary double-bind" for consideration for publication in Molecular Systems Biology.

We have now considered your manuscript and I regret to inform you that we have decided to not send it out for peer review.

In this study, you screened candidate drugs at a range of increasing drug concentrations for potential synergistic combinations with chemotherapy in MCF-7 and T-47D cell lines that had been evolved to have resistance to two different chemotherapy drugs, paclitaxel and doxorubicin. We appreciate that you report disulfiram treatment has a synergistic effect with chemotherapy in doxorubicin-resistant MCF-7 cells and that a simultaneous combination treatment was predicted and validated to be superior to a sequential alternating treatments. Unfortunately we are not convinced that the study provides the kind of decisive advance and broadly relevant systems-level biological insights that would be required for publication in Molecular Systems Biology. In addition, the novelty of the findings on disulfiram synergism in MCF-7 cells is somewhat limited by Lafi et al. 2023 Plos One.

That said, your work is a good candidate for Life Science Alliance (http://www.life-science-alliance.org/) our broad scope Open Access journal published in partnership between EMBO Press, Rockefeller University Press, and Cold Spring Harbor Laboratory Press. The editors of Life Science Alliance would be pleased to send your manuscript for peer review. Please use the following link to transfer your manuscript (no reformatting is required): Link Not Available

Eric Sawey, executive editor of Life Science Alliance (e.sawey@life-science-alliance.org), would be happy to answer any questions.

I apologize for not bringing better news regarding the publication of your study in Molecular Systems Biology and I hope that you will view the possibility of a transfer to Life Science Alliance favorably.

Yours sincerely,

Poonam Bheda, PhD
Scientific Editor
Molecular Systems Biology

==========================

** As a service to authors, EMBO Press offers the possibility to directly transfer declined manuscripts to another EMBO Press title or to the open access journal Life Science Alliance launched in partnership between EMBO Press, Rockefeller University Press and Cold Spring Harbor Laboratory Press. The full manuscript and if applicable, reviewers' reports, are automatically sent to the receiving journal to allow for fast handling and a prompt decision on your manuscript. For more details of this service, and to transfer your manuscript please click on Link Not Available. **

Dear Dr. Bheda,

Thank you for considering our manuscript entitled “A novel therapy for ER+ breast cancer suppresses drug resistance via an evolutionary double-bind” for publication in Molecular Systems Biology. We are grateful for the time and effort that you have dedicated toward reviewing our research and your valuable comments have been taken into account.

We are writing to clarify some of the points raised in your decision regarding our paper in hope that this information may be considered.

We were motivated to submit our manuscript to Molecular Systems Biology as your journal publishes research that aims to explain the properties and behavior of complex and dynamic biological systems along with the integration of large datasets with computational models. As previous work published in this journal has explored ecological and evolutionary principles and mathematical modeling to better understand cancer (Larsson et al., 2021; Park and Lehner, 2015; Nelander et al., 2008), our study builds upon these concepts while emphasizing the application of evolutionary game theory, which describes the use of mathematical models of strategic interactions to study competition in evolving populations (i.e., drug resistant and sensitive cells). Therefore, we are excited by the focus of MSB, and believe our work falls within the scope of the journal, as our work covers the evolution of dynamic biological networks along with the integration of computational biology.

Additionally, our findings have prospective impact in the field as we investigate the potential of exploiting an “evolutionary double-bind”, where treatment with one drug improves the response to a different agent. Our work identifies a treatment double-bind in ER+ breast cancer and proposes an experimental-mathematical framework with broad applicability in designing evolution-based or adaptive therapy treatment strategies which may be applied across a range of cancer types, thus highlighting the translational value of our findings.

Finally, regarding the novelty of our work and the finding limitations based on the referenced Lafi et al. 2023 PLOS One article on disulfiram synergism, there are significant differences which set our work apart from Lafi et al. (in experimental execution, novelty, and impact of findings). While both bodies of work present chemo re-sensitization involving a combination treatment with disulfiram, our research differs in that we introduce evolutionary game theory mathematical applications which validate treatment effects given heterogenous populations and growth dynamics. To thoroughly examine eco-evolutionary interactions and the corresponding treatment impacts in our system, we conducted long-term experiments of 5 weeks (as opposed to 72 hours in Lafi et al.), using 3D co-cultured cells plated at various ratios (versus 2D, monocultured cells in Lafi et al.), and various treatment schedules (continuous, sequential, and staggered whereas Lafi et al. uses a one-

time treatment administration). This comprehensive dataset was then used to develop, parameterize, and test our model which ultimately assessed the best strategy for reducing the selection of resistant populations. Additionally, Lafi et al. introduces a third drug, Hydrazine, which has conflicting clinical results regarding its efficacy in cancer, while suggesting it may, itself, be a carcinogen as it promotes some cancers [PMID: 26389475]. Collectively, we feel our work offers novel broad system level insights beyond those presented in Lafi et al. that are appropriate for publication in Molecular Systems Biology.

We hope these points allow a reconsideration for moving forward in the peer review process due to the manuscript's relevance to the journal's scope, potential impact in the field, and the novelty and quality of our research.

Once again, we thank you for your time and thoughtful review of our manuscript, and we remain open for any further questions or advisement with regards to proceeding forward.

On behalf of the authors, sincerely yours,

Andrea Bild, Ph.D.

Professor

Division of Molecular Pharmacology

Department of Medical Oncology & Therapeutics Research

City of Hope

1218 South 5th Avenue, Monrovia CA 91016

e-mail: abild@coh.org

19th Mar 2025

Manuscript Number: MSB-2025-12854R-Q
Title: A novel therapy for ER+ breast cancer suppresses drug resistance via an evolutionary double-bind

Dear Dr. Bild,

Thank you for the submission of your manuscript to Molecular Systems Biology. We have now received feedback from the three reviewers who agreed to evaluate your manuscript. As you will see from the reports below, the referees acknowledge the interest of the study and are overall supportive of your work; however they also comment on parts of the manuscript that should be strengthened in a revision. I think the reviews of Reviewer 1 and 2 are quite clear, and I will not repeat their comments. One important aspect to improve according to Reviewer 3 would be clarify and highlight the significance of the study and whether/how the modeling revealed new biological insights and could be applied to other systems and scenarios. All other issues raised would need to be satisfactorily addressed. Please let me know in case you would like to discuss in further detail any of the comments, I would be happy to schedule a call.

When submitting your revised manuscript, please carefully review the instructions that follow below. We perform an initial quality control of all revised manuscripts before re-review; failure to include requested items will delay the evaluation of your revision.

We require:

1) A .docx formatted version of the manuscript text (including legends for main figures, EV figures and tables). Please make sure that the changes are highlighted to be clearly visible. Alternatively you may choose to submit your manuscript as a LaTeX file.

2) Individual production quality figure files as .eps, .tif, .jpg (one file per figure). For guidance, download the 'Figure Guide PDF' (https://www.embopress.org/page/journal/17574684/authorguide#figureformat).

3) At EMBO Press we ask authors to provide source data for the main figures. Our source data coordinator will contact you to discuss which figure panels we would need source data for and will also provide you with helpful tips on how to upload and organize the files.

4) A .docx formatted letter INCLUDING the reviewers' reports and your detailed point-by-point responses to their comments. As part of the EMBO Press transparent editorial process, the point-by-point response is part of the Peer Review File (PRF), which will be published alongside your paper.

5) A complete author checklist, which you can download from our author guidelines (https://www.embopress.org/page/journal/17574684/authorguide#submissionofrevisions). Please insert information in the checklist that is also reflected in the manuscript. The completed author checklist will also be part of the PRF.

6) Please note that all corresponding authors are required to supply an ORCID ID for their name upon submission of a revised manuscript.

7) It is mandatory to include a 'Data Availability' section after the Materials and Methods. Before submitting your revision, primary datasets produced in this study need to be deposited in an appropriate public database, and the accession numbers and database listed under 'Data Availability'. Please remember to provide a reviewer password if the datasets are not yet public (see https://www.embopress.org/page/journal/17574684/authorguide#dataavailability).

In case you have no data that requires deposition in a public database, please state so in this section as follows: "This study includes no data deposited in external repositories". Note that the Data Availability Section is restricted to new primary data that are part of this study.

8) All Materials and Methods need to be described in the main text using our 'Structured Methods' format, which is required for all research articles. According to this format, the Methods section includes a Reagents and Tools Table (listing key reagents, experimental models, software and relevant equipment and including their sources and relevant identifiers) followed by a Methods and Protocols section describing the methods using a step-by-step protocol format. The aim is to facilitate adoption of the methodologies across labs. Please upload the Reagents and Tools table as a separate document when submitting your revised manuscript. More information on how to adhere to this format as well as a downloadable template (.docx) for the Reagents and Tools Table can be found in our author guidelines:
https://www.embopress.org/page/journal/17444292/authorguide#structuredmethods

An example of a Method paper with Structured Methods can be found here:
https://www.embopress.org/doi/10.15252/msb.20178071.

9) For data quantification: please specify the name of the statistical test used to generate error bars and p-values, the number (n) of independent experiments (specify technical or biological replicates) underlying each data point and the test used to calculate p-values in each figure legend. The figure legends should contain a basic description of n, p-values and the test applied. Graphs must include a description of the bars and the error bars (s.d., s.e.m.). Please provide exact p-values (in either the figure or figure legend).

10) Our journal encourages inclusion of *data citations in the reference list* to directly cite datasets that were re-used and obtained from public databases. Data citations in the article text are distinct from normal bibliographical citations and should directly link to the database records from which the data can be accessed. In the main text, data citations are formatted as follows: "Data ref: Smith et al, 2001" or "Data ref: NCBI Sequence Read Archive PRJNA342805, 2017". In the Reference list, data citations must be labeled with "[DATASET]". A data reference must provide the database name, accession number/identifiers and a resolvable link to the landing page from which the data can be accessed at the end of the reference. Further instructions are available at .

11) We replaced Supplementary Information with Expanded View (EV) Figures and Tables that are collapsible/expandable online. EV Figures should be cited as 'Figure EV1, Figure EV2" etc... in the text and their respective legends should be included in the main text after the legends of regular figures.
- For the figures that you do NOT wish to display as Expanded View figures, they should be bundled together with their legends in a single PDF file called *Appendix*, which should start with a short Table of Content. Appendix figures should be referred to in the main text as: "Appendix Figure S1, Appendix Figure S2" etc.
- Additional Tables/Datasets should be labeled and referred to as Table EV1, Dataset EV1, etc. Legends should be provided in a separate tab in case of .xls files. Alternatively, the legend can be supplied as a separate text file (README) and zipped together with the Table/Dataset file.
See detailed instructions here:
https://www.embopress.org/page/journal/17574684/authorguide#expandedview

12) Author contributions: CRediT has replaced the traditional author contributions section because it offers a systematic machine-readable author contributions format that allows for more effective research assessment. Please remove the Authors Contributions from the manuscript and use the free text boxes beneath each contributing author's name in our system to add specific details on the author's contribution. More information is available in our guide to authors.

13) Disclosure statement and competing interests: We updated our journal's competing interests policy in January 2022 and request authors to consider both actual and perceived competing interests. Please review the policy https://www.embopress.org/competing-interests and update your competing interests if necessary.

14) Every published paper now includes a 'Synopsis' to further enhance discoverability. Synopses are displayed on the journal webpage and are freely accessible to all readers. They include a short stand first (maximum of 300 characters, including space) as well as 2-5 one-sentences bullet points that summarizes the paper. Please write the bullet points to summarize the key NEW findings. They should be designed to be complementary to the abstract - i.e. not repeat the same text. We encourage inclusion of key acronyms and quantitative information (maximum of 30 words / bullet point). Please use the passive voice. Please attach these in a separate file or send them by email, we will incorporate them accordingly.

Please also suggest a striking image or visual abstract to illustrate your article as a PNG file 550 px wide x 300-600 px high.

Please note that these would be the final versions and changes during proofing are usually not allowed.

15) As part of the EMBO Publications transparent editorial process initiative (see our policy here: https://www.embopress.org/transparent-process#Review_Process), Molecular Systems Biology will publish online a Peer Review File (PRF) to accompany accepted manuscripts.
In the event of acceptance, this file will be published in conjunction with your paper and will include the anonymous referee reports, your point-by-point response and all pertinent correspondence relating to the manuscript. Let us know whether you agree with the publication of the PRF and as here, if you want to remove or not any figures from it prior to publication.
Please note that the Author checklist will be published at the end of the PRF.

Molecular Systems Biology has a "scooping protection" policy, whereby similar findings that are published by others during review or revision are not a criterion for rejection. Should you decide to submit a revised version, I do ask that you get in touch after three months if you have not completed it, to update us on the status.

I look forward to receiving your revised manuscript.

Yours sincerely,

Poonam Bheda, PhD

Scientific Editor
Molecular Systems Biology

Reviewer #1:

The evolution of resistance, clearly an evolutionary process, is rarely analysed using tools form evolutionary dynamics. Therefore the combination of a mathematical model and experiments reported in this manuscript is welcome - I wish there were more papers like this. The text is well written in general, but there are some parts that I wish were written more clearly. And I have a minor issue with the theory. But these are minor points that do not impair the value of the paper.

Abstract:
- "ER+" in the abstract is not appropriate - what is it?
- The definition of double bind in the abstract is inaccurate and unclear: strictly speaking, it is not that one treatment improves the other - rather it is a reduced likelihood of developing resistance to two simultaneous treatments. The definition in the introduction (below) is more rigorous.
- "A dose-dependent re-sensitization (a double-bind)": is this the definition of the double bind? It is unclear. The definition in the introduction ("whereby..." at the end of first paragraph) is better
- Evolutionary models, or any models, are not "used in refractory cancers", or any cancers... they are used to analyse cancer...
- "reducing the selection of resistant populations" is unclear. Do you mean reduce the evolution of resistance?
- "develop and parametrize a model form the data": one doesn't develop models from data - parametrize yes.
- The final part mentioning "selection for resistance to chemotherapy induces sensitivity to disulfiram" sounds intriguing but the abstract is so badly written that it is not enticing (and after reading the rest of the manuscripit, I find it surprisingly sloppy and badly written compared to the rest of the text, which is in general clear and well written).

Intro
- The definition in the introduction (end of first paragraph: "whereby...") is ok
- The focus here seems to be on (finding synergies for) cancers with evolved resistance (there is already work about synergies for first-line treatment). I don't know much about the field, so I wonder: why not use the same methods used for first-line treatments? what is the difference with the methods used here?
- "They do not address potential cell-cell interactions": what does it mean? How is that a problem for the assays mentioned there (collateral sensitivity assays do provide insight
into drug interactions that lead to increased efficacy when in combination)? The example that follows doesn't help understand. It only says that cells interact. Of course cells interact in the ways mentioned, but it's not clear why "cell-cell interactions during the drug screening process ... aid in determining the robustness of drug synergy within heterogeneous tumors".
- EGA: measuring growth rates of populations of different fractions of the two types: how does that help the assay (collateral sensitivity assays do provide insight into drug interactions that lead to increased efficacy when in combination)?
- I don't understand the rationale (it is not explained) for the four characteristics hypothesised for an ideal secondary drugs. Of course the two drugs must be synergistic and remain so in the presence of evolved resistance. And, likewise, of course "the secondary drug would preferentially target evolved-resistance cells while simultaneously leading to total tumor regression". I don't understand how these four points help - they seem obvious. Do they actually help select the best treatment later in the results?
- The rationale for exploring scheduling regimes, instead, at the end of the introduction, is clear and makes sense...
- ...but I don't understand... now you say you are going to model and test adaptive therapy (sensu Gatenby) rather than the double-bind?

Screening for candidate evolutionary double-bind drugs in ER+ breast cancer
- Define "ER+"
- This is an interesting and exciting summary of what's going to be revealed next.
- But "we classified it as an evolutionary double-bind due to metrics of 1) drug-drug synergy and 2) sensitive-resistant cell-cell interactions" is unclear

Results
- Candidate drugs screened for potential synergistic combinations: The part about identifying disulfiram is ok, clear.
- Quantifying collateral sensitivity: impressive set of results, but can you clarify why the effects are mostly neutral (how does that follow from the figures?) and what is special about figure 2C? I don't see how it differs from the other panels, and just saying that MUSYC predicts that doesn't help. I just don't understand why you conclude the effect is additive (I am not questioning that it is - I just don't think it's been explained properly, at least not in a way that I can grasp). I also don't understand your discussion about "additional stabilizing effect - does it meant that the "bifurcated effect" disappears?
- Quantifying cell-cell interactions: this section seems ok, I have no issues. Now I think this is the part you referred to as "cell-cell interactions", and it makes sense.

- Classifying selection dynamics: Why isn't there an equivalent of Figure 3 B, D F, H for the case in Figure 4C? That's the one with some more diverse dynamics (middle panel) It would help see the dynamics because, to be rigorous, checking only the invasion conditions in pure populations does not rule out the existence of mixed equilibria. Showing, at least, that the two fitness functions do not intersect, would suggest that bistability and coexistence do not happen. And actually, since bistability and coexistence do not seem possible (given the linear dynamics) are the middle panels necessary?
- Evolutionary Game Assay Validation ok.

Apart from these minor issues, I enjoyed reading the manuscript, thought I had a hard time understanding everything the first time I read it. I hope the comments above are helpful. This is the first time, I believe, I read of a proper analysis of a case of double bind, and the combination of evolutionary modelling and experiments is commendable.

Reviewer #2:

This study explores a novel strategy to combat chemotherapy resistance in ER+ metastatic breast cancer by identifying an "evolutionary double-bind," whereby resistance to one drug creates sensitivity to another. The work here focuses on disulfiram, an FDA-approved anti-alcoholism drug, as a potential drug candidate.

Disulfiram effectively inhibited the survival of ER+ chemotherapy- resistant cells, suggesting it exploits vulnerabilities arising from drug resistance. An integrated mathematical-experimental framework is described, combining in vitro measurements with game-theoretic modeling, to quantify drug synergy and cell-cell interactions.
Evolutionary game assays (EGA) measure the frequency-dependent growth of sensitive and resistant cells under various treatments, demonstrating that the combination of disulfiram and chemotherapy results in decreased tumor fitness. A combination therapy regimen is shown to outperform sequential administration.

The study is compelling in highlighting a new workflow for drug repurposing studies, which may improve the time and cost of drug development and offer personalized therapeutic potential.

Major Comments

The main question that arises from these interesting results is: what is known about the molecular specifics of the underlying resistance mechanisms? And are there commonalities that can be identified? In the discussion, the authors notes that this will be the basis of future studies. Setting aside that major question for future work, the following points could be addressed within this study:

1. Please address how similar are the initial resistance properties that emerge in biological replicate cultures of the same cell line with the same dox/paclitaxel long-term weekly dosing regimen. Although molecular characterization is planned for future work, can the authors speak to variation in the frequency with which replicate cultures display resistance, the timing of onset, whether there were outliers that did not develop resistance, etc. These observations may also impact how we think about the translational aspects of this work.

2. If I understand correctly, the dox and paclitaxel resistant lines are generated in standard culture conditions for the MCF7 and T-47D lines, and then the cells are shifted to mono and co-culture spheroids for subsequent studies. What is the rationale for performing the initial treatments in 2D? If the 3D culture more closely mimics the tumor behavior, would this not also apply to the initial generation of chemoresistance?

3. Is the lentiviral vector labeling the cell cytoplasm or nucleus? From the images in Figure 3, it appears that some treatments impact cell density (without much change in spheroid dimensions) while others do change the spheroid diameter. Can the authors comment? Is there a density limit beyond which it is not feasible to distinguish differences in spheroid cell number? How is this aspect dealt with?

4. Fig 4 is fairly complex and the authors could put some additional explanation statements into the legend to assist the reader with interpretation. Some aspects do not stand-alone without referring back to the "Quantifying cell-cell interactions" section of the results. For example the C-A / B-D abbreviations might best be de-noted as deltaS or deltaR, or else defined again somewhere within the legend. In addition, please check the grey color used for the untreated samples. Unless I misunderstand the scheme, there is a mismatch between the legend and data labels.

Reviewer #3:

I am sorry, if my response may sound a little bit dismissive. This is not my intention, and this is a serious discussion. It is of course possible that I have missed something.

It is widely known that disulfiram can exert anti-cancer effects. I just had a look at PubMed and 'disulfiram cancer' resulted in 825 hits, with the earliest reports being from the 1960s and 1970s. Given this long history, disulfiram does not appear to be a particularly strong candidate for clinical translation. If it was one, it would probably have long found its way into routine clinical use?

However, I appreciate that this study is not primarily focused on the identification of clinically effective drug candidates for translational studies. It is primarily about the development of better approaches for the identification of effective anti-cancer drug combinations. However, I do not really understand what all the (modelling) experiments actually add to the findings from the initial screen and the confirmation experiments. They basically confirm that disulfiram increases the efficacies of doxorubicin and paclitaxel in the investigated models? Or is there an additional benefit, a new approach that will us help to identify effective combination therapies faster in the future?

The third question that I have refers to the models that you used. Given the heterogeneity in cancer, including the unpredictable nature of cancer cell evolution in general and in response to anti-cancer drugs, the effects of disulfiram may be specific for the limited number of models that you used. In this context, it would be really interesting to check how specific your findings are in a broader range of experimental systems, i.e. using additional cell lines and additional drugs. Is there a way to use your findings in an individualized way? Or how would you anticipated that they can improve cancer therapies?

Moreover, I struggled with the figures, most probably, because I was not able to find the legends. In Figure 3(?, the cell lines grown together in spheres). The resistant cells are not growing? This makes me ask whether this is an appropriate model, as cancer cells that do not grow do not seem to be the main concern? Moreover, the effects of disulfiram and doxorubicin on the sensitive cells appears to be very limited? If you want to have an effective treatment, you will have to kill all cancer cells and not just select for 'sensitive' ones? Conceptually, this may be not of primary interest, but in a therapeutic context, it would be interesting to know whether the applied drug concentrations were within the range of clinical plasma levels.

Taken together, I do not see at the moment, how your approach may contribute to progress in the identification of combination therapies for cancer. It is also not clear to me what conceptual progress could result from your approach in terms of a better understanding of the processes underlying the combined effects of anti-cancer drugs. Given that disulfiram has been investigated for quite a while for the application that you are proposing and that it seems to be an unlikely candidate for clinical translation (I appreciate that I might regret this bold judgement in the future, if I am proven wrong), what is the main innovation that people should use to design better cancer therapies or to understand cancer therapies better?

Note to all reviewers

We thank all reviewers for their helpful comments & critiques, and have responded below in a point-by-point reply. For convenience, responses are color-coded for each reviewer, with corresponding changes in the manuscript main text indicated with the same color scheme:

Reviewer #1: purple
Reviewer #2: blue
Reviewer #3: green

Reviewer #1:

The evolution of resistance, clearly an evolutionary process, is rarely analysed using tools from evolutionary dynamics. Therefore the combination of a mathematical model and experiments reported in this manuscript is welcome - I wish there were more papers like this. The text is well written in general, but there are some parts that I wish were written more clearly. And I have a minor issue with the theory. But these are minor points that do not impair the value of the paper.

Inquiry/Comment #1: (Abstract) "ER+" in the abstract is not appropriate - what is it? Estrogen Receptor Positive

> **Response:** We thank the reviewer for their question. ER+ refers to Estrogen Receptor Positive. Breast cancer is commonly classified into hormone receptor subtypes based on the presence or absence of specific receptors. These classifications have important implications for prognosis and treatment. The main hormone-based classifications are Hormone Receptor-Positive (HR+), which is further divided into ER+ (Estrogen-Receptor-positive) and PR+ (Progesterone Receptor-positive), HER2+ (Human Epidermal Growth Factor Receptor 2), and Triple-negative (negative for ER, PR, and HER2). **Our work focuses on ER+.**
>
> However, we agree it is not appropriate to use an acronym without describing the full term in the abstract. This correction has been made in the manuscript.

Inquiry/Comment #2: (Abstract) The definition of double bind in the abstract is inaccurate and unclear: strictly speaking, it is not that one treatment improves the other

- rather it is a reduced likelihood of developing resistance to two simultaneous treatments. The definition in the introduction (below) is more rigorous.

> **Response:** Thank you for bringing this to our attention. We have clarified the definition of a "double-bind" in order to achieve a clear understanding of the term across different sections of the manuscript. In the abstract, the definition of the "evolutionary double-bind" reads "where adapting to one treatment inadvertently makes them more susceptible to another treatment". We hope this definition edit provides clarity and consistency throughout the manuscript.

Inquiry/Comment #3: (Abstract) "A dose-dependent re-sensitization (a double-bind)": is this the definition of the double bind? It is unclear. The definition in the introduction ("whereby..." at the end of first paragraph) is better

> **Response:** We thank the reader for pointing out this confusion. We have removed "a double-bind" from this sentence as it was meant to imply the re-sensitization is possible *via* a double-bind. The definition of a double-bind is referenced elsewhere in the manuscript, and, upon further review, it appears unnecessary and redundant in this given sentence.

Inquiry/Comment #4: (Abstract) Evolutionary models, or any models, are not "used in refractory cancers", or any cancers... they are used to analyse cancer...

> **Response:** We have edited this sentence to provide clarity and improve readability.

Inquiry/Comment #5: (Abstract) "reducing the selection of resistant populations" is unclear. Do you mean reduce the evolution of resistance?

> **Response:** Our intention was to highlight the reduced selection of resistant populations as a means to prevent resistant cells from proliferating and/or becoming the dominant population. Cancer cells evolve under selective pressure from therapy, and reducing selection pressure in such a way would mean less chance for resistant clones to dominate. The ultimate goal is to discourage the emergence or expansion of resistant populations. The text has been edited with the hopes of providing that clarity.

Inquiry/Comment #6: (Abstract) "develop and parametrize a model form the data": one doesn't develop models from data - parametrize yes.

Response: We thank the reviewer for making this point. The text has been edited to reflect this.

Inquiry/Comment #7: (Abstract) The final part mentioning "selection for resistance to chemotherapy induces sensitivity to disulfiram" sounds intriguing but the abstract is so badly written that it is not enticing (and after reading the rest of the manuscript, I find it surprisingly sloppy and badly written compared to the rest of the text, which is in general clear and well written).

Response: Following the edits made in response to the reviewers' thoughtful comments above, we hope the readability of the abstract is improved, resulting in an increase in interest for the work to come.

Inquiry/Comment #8: (Introduction) The focus here seems to be on (finding synergies for) cancers with evolved resistance (there is already work about synergies for first-line treatment). I don't know much about the field, so I wonder: why not use the same methods used for first-line treatments? what is the difference with the methods used here?

Response: We appreciate the reviewer's question. While methods used for first-line synergy discovery have been valuable, our approach extends beyond them to account for the unique analytical and biological challenges posed by evolved resistance, where tumor ecology and population dynamics play a dominant role in shaping therapeutic outcomes.

Analytical methods used in first-line combination therapy screenings often assume relatively uniform tumor response. However, in resistant disease, treatment response is complicated by multi-drug resistance, diverse and overlapping resistance mechanisms, and heterogeneous tumor cell populations. Our work develops new analysis methods for assessing combination therapies using drug screening data that explicitly considers the impact of heterogeneous cell-cell interactions. These interactions can negate, enhance, or reshape drug synergy depending on the competitive or cooperative dynamics within the tumor. This ecological perspective is largely absent from traditional synergy assessment methods used in the context of first-line treatments. Our methods also consider collateral sensitivity, where evolved resistance to one drug induces sensitivity to another. This is underexplored, and investigating whether drug synergies emerge or change after resistance evolves remains an open question.

Therefore, our methods differ by introducing a novel experimental-mathematical framework that integrates evolutionary game theory with long-term 3D co-culture data to identify effective drug combinations in the context of acquired chemoresistance.

Inquiry/Comment #9: (Introduction) "They do not address potential cell-cell interactions": what does it mean? How is that a problem for the assays mentioned there (collateral sensitivity assays do provide insight into drug interactions that lead to increased efficacy when in combination)? The example that follows doesn't help understand. It only says that cells interact. Of course cells interact in the ways mentioned, but it's not clear why "cell-cell interactions during the drug screening process ... aid in determining the robustness of drug synergy within heterogeneous tumors".

Response: We appreciate the reviewer's important comment. We agree that clarification is needed regarding the distinction we are making between traditional collateral sensitivity assays and the inclusion of cell-cell interactions in assessing drug synergy, especially in the context of heterogeneous tumors. Many traditional collateral sensitivity drug assays are predominantly conducted using monocultures due to their simplicity and suitability for high-throughput screening. However, there is a growing interest in incorporating co-cultured heterogeneous cell populations to better mimic the complexity of tumor environments and to capture the diverse responses of cancer cells to therapeutic agents. Responses might change when multiple cell types co-exist and interact, as they would in actual tumors. In a tumor composed of drug-sensitive and drug-resistant subpopulations, the fitness and drug response of each population can be influenced by the presence and behavior of the other. Such interactions can dampen or enhance the effectiveness of a drug combination compared to what would be predicted from monoculture-based synergy assays. Thus, when we state that "they do not address potential cell-cell interactions," we are referring to the inability of traditional assays to account for these ecological effects. To address this, our study integrates evolutionary game theory and the Evolutionary Game Assay (EGA), which directly measures the frequency-dependent growth rates of sensitive and resistant populations in co-culture under various treatments. By doing so, we can capture how drug efficacy and synergy are influenced by population composition and interaction dynamics, providing a more robust and relevant understanding of combination treatment effects in heterogeneous tumors. Additionally, we go into greater detail on such "cell-cell interactions" in another section of the introduction (see ***"Quantifying cell-cell interactions"*** later in the introduction).

Inquiry/Comment #10: (Introduction) EGA: measuring growth rates of populations of different fractions of the two types: how does that help the assay (collateral sensitivity assays do provide insight into drug interactions that lead to increased efficacy when in combination)?

Response: See response to Inquiry/Comment #9 above. Also see ***"Quantifying cell-cell interactions"*** later in the introduction.

Inquiry/Comment #11: (Introduction) I don't understand the rationale (it is not explained) for the four characteristics hypothesised for an ideal secondary drugs. Of course the two drugs must be synergistic and remain so in the presence of evolved resistance. And, likewise, of course "the secondary drug would preferentially target evolved-resistance cells while simultaneously leading to total tumor regression". I don't understand how these four points help - they seem obvious. Do they actually help select the best treatment later in the results?

Response: Thank you for presenting this inquiry. To clarify, the four hypothesized characteristics, while they may appear obvious, were intended to define specific, testable criteria that guided both our experimental design and the selection of a secondary drug (from our screen) in the context of acquired resistance.

While strong synergy is obviously essential, it is not always sufficient as synergistic effects can degrade once resistance emerges. Thus, our second and third criteria focus on whether synergy is preserved or enhanced in both resistant monocultures and co-cultures of sensitive and resistant cells, which better reflects tumor heterogeneity. The fourth criterion, preferential targeting of resistant cells, addresses the evolutionary objective of reducing selection for resistance, while still achieving meaningful tumor control.

In the **Results**, we show how these principles were used to identify disulfiram as a candidate that meets all four criteria. Specifically, disulfiram maintained or enhanced synergy with chemotherapy among resistant lines, displayed preferential efficacy against resistant cells, and ultimately resulted in decreased resistant cell growth in co-culture models. Therefore, the framework provided a structured basis for evaluating and prioritizing drug combinations.

Inquiry/Comment #12: (Introduction) The rationale for exploring scheduling regimes, instead, at the end of the introduction, is clear and makes sense...but I don't

understand... now you say you are going to model and test adaptive therapy (sensu Gatenby) rather than the double-bind?

Response: Thank you for the question. We do not address adaptive therapy anywhere within the manuscript or the mathematical modeling. But we do reference the Gatenby paper and other “evolution-based” treatment strategies in the Introduction, as alternative approaches. We have reworded this sentence for clarity.

Inquiry/Comment #13: (Introduction) Screening for candidate evolutionary double-bind drugs in ER+ breast cancer - Define "ER+"

Response: We thank the reviewer for drawing our attention to this error. The text has been edited to define ER+.

Inquiry/Comment #14: (Introduction) But "we classified it as an evolutionary double-bind due to metrics of 1) drug-drug synergy and 2) sensitive-resistant cell-cell interactions" is unclear

Response: We appreciate the opportunity to clarify this statement.

By referring to "metrics of 1) drug-drug synergy and 2) sensitive-resistant cell-cell interactions," we aimed to define quantifiable criteria for identifying an evolutionary double-bind. We found disulfiram in combination with chemotherapy to have met the criteria in the following ways.

1. *Drug-drug synergy*: We assessed whether the combination of the first-line chemotherapy and the secondary drug (in this case, disulfiram) produced a greater effect together than expected based on their individual activities—both in resistant cell monocultures and in mixed populations. This indicates that the drugs are not merely additive but interact in a way that supports synergy.
2. *Sensitive-resistant cell-cell interactions*: We used evolutionary game assays (EGA) to measure how the presence of resistant cells affects the growth of sensitive cells (and vice versa) under different treatments. This allowed us to determine whether disulfiram selectively creates a disadvantage for resistant cells, especially when they are competing with sensitive ones. Through our experiments/EGA, we were able to conclude that resistance to the first drug reduced fitness in the presence of the second.

Taken together, the data supports our identification of an evolutionary double-bind. We have revised the manuscript to clarify this rationale and thank the reviewer for highlighting the ambiguity.

Inquiry/Comment #15: (Results) Quantifying collateral sensitivity: impressive set of results, but can you clarify why the effects are mostly neutral (how does that follow from the figures?) and what is special about figure 2C? I don't see how it differs from the other panels, and just saying that MUSYC predicts that doesn't help. I just don't understand why you conclude the effect is additive (I am not questioning that it is - I just don't think it's been explained properly, at least not in a way that I can grasp). I also don't understand your discussion about "additional stabilizing effect - does it meant that the "bifurcated effect" disappears?

Response: This is an excellent question, and we now address the key parameters in the MUSYC framework and their relationship to synergy and antagonism. We also include this schematic explainer in the Expanded View Material (**Figure EV2**).

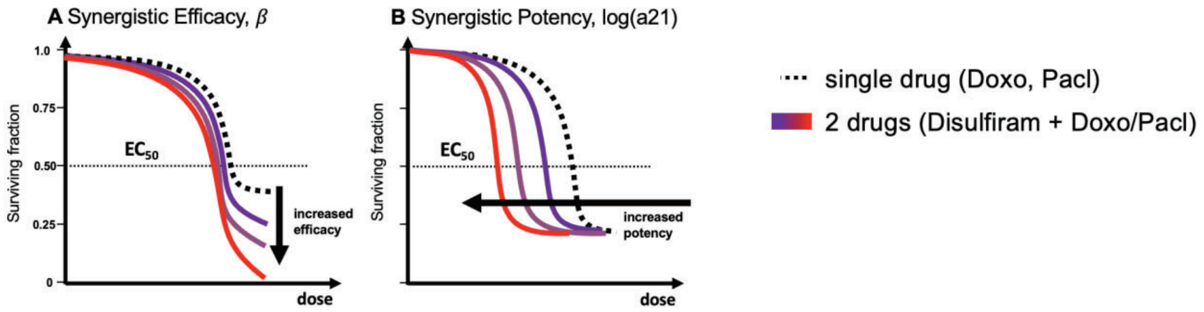

Regarding the bifurcation effect, the Reviewer is correct that the bifurcated effect in figure 2B,C,D (high doses of disulfiram) disappears under combination with doxorubicin. We have reworded in the manuscript for clarity.

> *"While intermediate doses (eg 10^{-4}, 10^{-5}) achieve increasing response, higher doses (eg 10^{-3}) of disulfiram result in less response, causing the dose response curve to be non-monotonic. This observed effect disappears when disulfiram is given in combination with chemotherapy."*

Inquiry/Comment #16: (Results) Classifying selection dynamics: Why isn't there an equivalent of Figure 3 B, D F, H for the case in Figure 4C? That's the one with some more diverse dynamics (middle panel) It would help see the dynamics because, to be

rigorous, checking only the invasion conditions in pure populations does not rule out the existence of mixed equilibria. Showing, at least, that the two fitness functions do not intersect, would suggest that bistability and coexistence do not happen. And actually, since bistability and coexistence do not seem possible (given the linear dynamics) are the middle panels necessary?

> **Response:** This is an excellent suggestion, and we include the corresponding time-dynamics and fitness functions for all cases of Figure 4 in the Expanded View material (**Figure EV3 - EV8**). We also would like to draw the reviewer's attention to the error bars in the middle panels of Figure 4A-C, indicating the statistical significance of the Selection Dynamics quadrant.

Reviewer #2:

This study explores a novel strategy to combat chemotherapy resistance in ER+ metastatic breast cancer by identifying an "evolutionary double-bind," whereby resistance to one drug creates sensitivity to another. The work here focuses on disulfiram, an FDA-approved anti-alcoholism drug, as a potential drug candidate.

Disulfiram effectively inhibited the survival of ER+ chemotherapy- resistant cells, suggesting it exploits vulnerabilities arising from drug resistance. An integrated mathematical-experimental framework is described, combining in vitro measurements with game-theoretic modeling, to quantify drug synergy and cell-cell interactions. Evolutionary game assays (EGA) measure the frequency-dependent growth of sensitive and resistant cells under various treatments, demonstrating that the combination of disulfiram and chemotherapy results in decreased tumor fitness. A combination therapy regimen is shown to outperform sequential administration.

The study is compelling in highlighting a new workflow for drug repurposing studies, which may improve the time and cost of drug development and offer personalized therapeutic potential.

Major Comments

The main question that arises from these interesting results is: what is known about the molecular specifics of the underlying resistance mechanisms? And are there commonalities that can be identified? In the discussion, the authors notes that this will

be the basis of future studies. Setting aside that major question for future work, the following points could be addressed within this study:

<u>Inquiry/Comment #1:</u> Please address how similar are the initial resistance properties that emerge in biological replicate cultures of the same cell line with the same dox/paclitaxel long-term weekly dosing regimen. Although molecular characterization is planned for future work, can the authors speak to variation in the frequency with which replicate cultures display resistance, the timing of onset, whether there were outliers that did not develop resistance, etc. These observations may also impact how we think about the translational aspects of this work.

> **<u>Response:</u>** We thank the reviewer for raising this important point regarding the reproducibility and variability of resistance phenotypes across biological replicates during long-term chemotherapeutic exposure.
>
> In our study, doxorubicin- and paclitaxel-resistant cell lines were developed using a standardized weekly 24-hour chemotherapy exposure protocol over a 6–8 month period in both MCF-7 and T-47D ER+ breast cancer cell lines. Across biological replicates, we observed consistent acquisition of phenotypic resistance, confirmed via shifts in dose–response curves and sustained viability at specific therapeutic drug concentrations. The majority of replicate cultures within cell lines displayed robust resistance by the end of the treatment period. Across the different cell lines, we did observe some variability in the rate of onset and degree of resistance. Specifically, certain replicates exhibited a delayed emergence of resistance or required a more gradual dose escalation. However, we did not encounter complete outliers that failed to develop resistance under the given conditions.
>
> Although molecular characterization of resistance mechanisms in these lines is ongoing and outside the scope of the current work presented, prior studies have demonstrated that resistance to doxorubicin and paclitaxel can arise through multiple distinct pathways—including upregulation of efflux pumps (e.g., *ABCB1*), modulation of apoptotic signaling (e.g., *BCL2*, *TP53*), activation of EMT programs, or enhanced DNA repair capacity—depending on both intrinsic and extrinsic factors (Gottesman et al., 2002; Holohan et al., 2013; Liu et al., 2016). Furthermore, work using single-cell and lineage tracing approaches has shown that such variation may be driven by clonal diversity, epigenetic adaptation, or stochastic processes (Sharma et al., 2010; Shaffer et al., 2017; Hata et al., 2016). In the current work, we present future aims that will elucidate mechanisms conferring disulfiram resistance in hopes to complement what is/will be known of

chemotherapy resistance mechanisms for effective targeting with synergistic drug combinations.

These observations reinforce the translational relevance of our experimental framework. Tumor resistance in patients likewise evolves through heterogeneous trajectories. By modeling resistance acquisition across multiple replicates and subsequently studying these populations in 3D co-culture spheroids, we are better able to simulate and capture the ecological complexity of resistant and sensitive tumor cell interactions. Our evolutionary game theory (EGT) framework further allows us to quantify these dynamics independently of specific molecular drivers, making it adaptable to a range of resistance phenotypes and application of a variety of drug schemas even beyond disulfiram. This robustness is particularly relevant to clinical translation, where treatment strategies must account for mechanistic heterogeneity.

Inquiry/Comment #2: If I understand correctly, the dox and paclitaxel resistant lines are generated in standard culture conditions for the MCF7 and T-47D lines, and then the cells are shifted to mono and co-culture spheroids for subsequent studies. What is the rationale for performing the initial treatments in 2D? If the 3D culture more closely mimics the tumor behavior, would this not also apply to the initial generation of chemoresistance?

Response: We thank the reviewer for their insightful question. The chemoresistant lines were generated under standard 2D culture conditions, after which they were introduced into 3D mono- and co-culture spheroid models for further experimentation. This approach was chosen for several practical and biological reasons.

The development of stable chemoresistant lines requires long-term, repeated drug exposure (over a 6–8 month period, as described in the Methods section). This process is most effectively and reproducibly conducted in 2D monolayer culture, where conditions such as drug exposure, nutrient availability, and cell density can be more easily controlled. In 2D, we can also effectively monitor and confirm the development of resistance quickly through standardized assays such as dose–response curves and viability measurements. Lastly, culturing in 2D allows for more reliable long-term maintenance and steady expansion of the cell lines, whereas 3D possesses temporal and spatial constraints that, while not considerable in a 4-5 week drug assay, would have greatly slowed the timeframe for generating such resistant cell lines.

Once stable resistance was established and validated, cells were transitioned into 3D spheroid culture to better recapitulate the tumor microenvironment and to explore the ecological and evolutionary interactions between sensitive and resistant populations. For our experimental needs relevant to the current work, the 3D spheroid structure is crucial for modeling spatial constraints, cell-cell interactions, and frequency-dependent fitness dynamics, which are central to our evolutionary game theory framework.

Importantly, our objective was to simulate the clinically relevant scenario where resistance has already emerged — mimicking tumors that have progressed on chemotherapy — and to understand how modeling selection dynamics under different drug schemas could impact the long term trajectory of resistant populations. By first establishing resistance in 2D and then studying the ecological dynamics in 3D, we were able to decouple resistance acquisition from the subsequent evolutionary interactions in heterogeneous environments, allowing clearer interpretation of treatment strategies such as the evolutionary double-bind.

Inquiry/Comment #3: Is the lentiviral vector labeling the cell cytoplasm or nucleus? From the images in Figure 3, it appears that some treatments impact cell density (without much change in spheroid dimensions) while others do change the spheroid diameter. Can the authors comment? Is there a density limit beyond which it is not feasible to distinguish differences in spheroid cell number? How is this aspect dealt with?

Response: We thank the reviewer for these important observations and questions.

The fluorescent proteins used for lentiviral labeling in our study (Venus and mCherry, delivered via LeGO-V2 and LeGO-C2 vectors, respectively) localize to the cytoplasm, not the nucleus. This cytoplasmic labeling facilitates high-resolution intensity-based quantification of cell populations using fluorescence microscopy during 3D spheroid imaging (see Methods: *Lentiviral labeling of sensitive and resistant cells*).

Regarding the reviewer's observation on spheroid size versus density, we agree and have noted similar phenomena. Some treatments, such as combination chemotherapy and disulfiram, lead to substantial decreases in fluorescence signal intensity (indicative of decreased cell number and/or viability of each cell population) without proportionate changes in spheroid diameter (which speaks to

the total composition of sensitive-resistant populations). In contrast, other treatments affect both spheroid dimensions and cell growth more uniformly. This likely reflects differences in how treatments influence cell packing density, cell viability, and matrix remodeling within the spheroid, consistent with prior reports showing that size alone can underestimate treatment efficacy in dense or necrotic spheroids (Nunes et al., 2019; Vinci et al., 2012).

To address this, we employed a **validated fluorescence intensity-based cell count model** for quantifying spheroid composition (see Expanded View Figure EV1 and Methods: *Cell number quantification*). We constructed standard curves correlating fluorescence intensity to absolute cell number across a range of initial densities and sensitive-to-resistant cell ratios. These calibrations were performed under conditions that include high-density spheroids to ensure that non-linearity at saturation levels could be accounted for.

We acknowledge that at very high cell densities, especially beyond ~250,000 cells per spheroid, the signal can approach saturation, potentially limiting precise quantification. To mitigate this, we optimized imaging conditions (e.g., exposure time, gain settings) and tracked both **spheroid area** and **fluorescence intensity** to distinguish between changes in size versus internal cell number, providing a multidimensional readout of treatment effect.

This dual-parameter approach (area + fluorescence-based cell count) allows us to more accurately assess treatment effects even in morphologically similar spheroids and enhances the robustness of our evolutionary game assay results.

4. Fig 4 is fairly complex and the authors could put some additional explanation statements into the legend to assist the reader with interpretation. Some aspects do not stand-alone without referring back to the "Quantifying cell-cell interactions" section of the results. For example the C-A / B-D abbreviations might best be de-noted as deltaS or deltaR, or else defined again somewhere within the legend. In addition, please check the grey color used for the untreated samples. Unless I misunderstand the scheme, there is a mismatch between the legend and data labels.

Response: Response: This is an excellent suggestion, and we have made modifications to Figure 4 for consistency and clarity. Additionally, we thank the reviewer for pointing out the inconsistency between the figure legend and panels in figure 4, great catch!

Reviewer #3:

<u>Inquiry/Comment #1:</u> I am sorry, if my response may sound a little bit dismissive. This is not my intention, and this is a serious discussion. It is of course possible that I have missed something. It is widely known that disulfiram can exert anti-cancer effects. I just had a look at PubMed and 'disulfiram cancer' resulted in 825 hits, with the earliest reports being from the 1960s and 1970s. Given this long history, disulfiram does not appear to be a particularly strong candidate for clinical translation. If it was one, it would probably have long found its way into routine clinical use?

> **<u>Response:</u>** We appreciate the reviewer's perspective and agree that disulfiram has previously been investigated as a potential anti-cancer agent. However, we would like to emphasize that despite what is currently presented in the literature, the clinical translation of disulfiram in oncology has remained limited not due to lack of potential efficacy, but primarily due to several contextual, mechanistic, and design-related barriers, many of which our current study helps to address in novel ways.
>
> For example, much of the early work on disulfiram's anti-cancer properties lacked a clearly defined measure of selective vulnerability. Our study is distinct in supporting disulfiram not just as a potentially effective agent, but as part of an evolutionary double-bind strategy (either in combination or sequential therapy), whereby resistance to chemotherapy induces a selective vulnerability to disulfiram (see **Discussion**). This positions disulfiram within a specific therapeutic context (in combination with chemotherapy in refractory ER+ breast cancer), enhancing its strategic value and providing us the opportunity to model its impact in heterogeneous populations over time.
>
> Regarding its candidacy for clinical translation, we would like to highlight that while disulfiram is not currently used as part of any routine oncology treatment regimen, this should not discourage the scientific community's interest in its therapeutic potential or preclude future clinical applications. Support for disulfiram as a promising therapy can be drawn from recent studies that show complexation with copper (DSF-Cu) significantly enhances its stability, potency, and specificity in preclinical models (Skrott et al., 2017; Liu et al., 2022). Furthermore, several clinical trials (e.g., NCT02671890, NCT05210374, NCT03323346) have revisited disulfiram, particularly in combination with metal ions or chemotherapeutic agents, underscoring renewed interest in disulfiram based on updated mechanistic insights and across various settings.

However, and importantly, we would like to clarify that the impact of our study is not limited to identifying disulfiram as a new drug candidate per se. Rather, the broader impact of the present work is the development and validation a novel experimental-mathematical framework for identifying and optimizing effective anti-cancer drug combinations, especially in the context of acquired chemoresistance.

Inquiry/Comment #2: However, I appreciate that this study is not primarily focused on the identification of clinically effective drug candidates for translational studies. It is primarily about the development of better approaches for the identification of effective anti-cancer drug combinations. However, I do not really understand what all the (modelling) experiments actually add to the findings from the initial screen and the confirmation experiments. They basically confirm that disulfiram increases the efficacies of doxorubicin and paclitaxel in the investigated models? Or is there an additional benefit, a new approach that will us help to identify effective combination therapies faster in the future?

Response: This is a reasonable question, and we will briefly restate the outline of the figures for clarity:

Figure 1: Screen for drugs that target chemo-resistance (**single drug, single cell line**)
Figure 2: Screen for drug-drug synergy (**multi-drug, single cell line**)
Figure 3: Screen for synergistic or antagnistic cell-cell interactions (**multi-drug, multi- cell line**)
Figure 4: Repeat of Figure 3 across cell line / drug permutations.
Figure 5: Test combination therapy versus intermittent therapy.

Traditionally, many published studies of drug screens are limited to measuring drug-drug synergy only, and neglect analysis of evolved resistance or cell-cell interactions between parental and resistant lines.

We have made several edits to the Introduction to more clearly explain the comprehensive nature of our approach. See also response to Inquiry #5.

Inquiry/Comment #3: The third question that I have refers to the models that you used. Given the heterogeneity in cancer, including the unpredictable nature of cancer cell evolution in general and in response to anti-cancer drugs, the effects of disulfiram may be specific for the limited number of models that you used. In this context, it would be really interesting to check how specific your findings are in a broader range of

experimental systems, i.e. using additional cell lines and additional drugs. Is there a way to use your findings in an individualized way? Or how would you anticipated that they can improve cancer therapies?

Response: We thank the reviewer for this important question. We agree that cancer is highly heterogeneous and that responses to therapy—including disulfiram—can vary across cancer types and models. However, we respectfully highlight that the goal of this study was not to exhaustively evaluate disulfiram across a wide panel of cancer models, but rather to establish and validate a novel experimental and mathematical framework for evaluating drug combinations in the context of acquired resistance. Our intent was to focus on two well-characterized ER+ breast cancer models (MCF-7 and T-47D) and two standard chemotherapeutic agents (doxorubicin and paclitaxel) and use this controlled system as a proof of concept for this framework.

Expanding the study to incorporate additional cell lines and drug combinations, while valuable in principle, poses significant practical and interpretative challenges. Our experimental design involved long-term resistance modeling, 3D co-culture spheroid assays, multiple drug scheduling regimens (continuous, sequential, staggered), and integration of evolutionary game theory modeling. This combination of experiments and modeling would have been difficult to scale to a larger panel of cell lines and drugs and may have diluted the depth and clarity we aimed to achieve within the scope of this current work. The core innovation of our study lies not in identifying disulfiram as a broadly effective anti-cancer agent, but in demonstrating how eco-evolutionary principles and cell-cell interaction dynamics can be incorporated into drug combination screenings and modeled to uncover effective treatment strategies that reduce selection for resistance.

Importantly, while disulfiram serves as an optimal example, the methodology we present is designed to be generalizable. The same framework could be applied in future studies to other cancer types, cell lines, and treatment options, etc., especially in the context of drug resistance. Our approach is also adaptable for use with patient-derived models, opening the door to more individualized treatment design.

In this light, we view our study as an initial demonstration of a scalable, evolution-informed strategy for evaluating and optimizing drug combinations. We hope this work will motivate further application of these methods across diverse systems, including personalized models, but we believe that such expansions are best

suited to follow-up studies rather than being incorporated into this foundational work.

Inquiry/Comment #4: Moreover, I struggled with the figures, most probably, because I was not able to find the legends. In Figure 3(?, the cell lines grown together in spheres). The resistant cells are not growing? This makes me ask whether this is an appropriate model, as cancer cells that do not grow do not seem to be the main concern? Moreover, the effects of disulfiram and doxorubicin on the sensitive cells appears to be very limited? If you want to have an effective treatment, you will have to kill all cancer cells and not just select for 'sensitive' ones? Conceptually, this may be not of primary interest, but in a therapeutic context, it would be interesting to know whether the applied drug concentrations were within the range of clinical plasma levels.

Response: We apologize and have now added legends to Figure 3.

Regarding the resistant growth: it is expected that resistant line will have slowed growth in untreated conditions when in co-culture with parental line due to a “cost of resistance”. You can observe that the resistant line grows in mono-culture (without parental cells) by ~an order of magnitude from 10^4 to 10^5 in 28 days in **Figure 3A, right panel**.

It is also expected that the resistant line will have slowed growth under disulfiram conditions, since the drug preferentially targets resistant cells.

We have also now added a line of zero growth to panels **3B, D, F, H** to guide the eye.

We have also now included 6 additional Expanded View Figures which illustrate all co-culture conditions for MCF-7 and T-47D growth across all treatment conditions, and the mathematical modeling fitness function best fit analysis.

Inquiry/Comment #5: Taken together, I do not see at the moment, how your approach may contribute to progress in the identification of combination therapies for cancer. It is also not clear to me what conceptual progress could result from your approach in terms of a better understanding of the processes underlying the combined effects of anti-cancer drugs. Given that disulfiram has been investigated for quite a while for the application that you are proposing and that it seems to be an unlikely candidate for clinical translation (I appreciate that I might regret this bold judgement in the future, if I

am proven wrong), what is the main innovation that people should use to design better cancer therapies or to understand cancer therapies better?

Response: We thank the reviewer for their critique, and have spent some time considering the major conceptual advances and progress that our manuscript contributes to the literature.

As we stated in our response to Inquiry #2, many published studies of drug screens are limited to measuring drug-drug synergy only (e.g. using MUSYC or similar frameworks), and neglect analysis of 1) evolved resistance or 2) cell-cell interactions between parental and resistant lines. Examples of this are myriad, but include the MUSYC framework:

1. Wooten, D.J., Meyer, C.T., Lubbock, A.L., Quaranta, V. and Lopez, C.F., 2021. MuSyC is a consensus framework that unifies multi-drug synergy metrics for combinatorial drug discovery. Nature communications, 12(1), p.4607.
2. Meyer, C.T., Wooten, D.J., Paudel, B.B., Bauer, J., Hardeman, K.N., Westover, D., Lovly, C.M., Harris, L.A., Tyson, D.R. and Quaranta, V., 2019. Quantifying drug combination synergy along potency and efficacy axes. Cell systems, 8(2), pp.97-108.

Alternatively, there have been other studies focused on measuring cell-cell interactions, but these typically neglect drug-drug synergy. Examples of this include:

1. Maltas, J., Tadele, D.S., Durmaz, A., McFarland, C.D., Hinczewski, M. and Scott, J.G., 2024. Frequency-dependent ecological interactions increase the prevalence, and shape the distribution, of preexisting drug resistance. PRX Life, 2(2), p.023010.
2. Farrokhian, N., Maltas, J., Dinh, M., Durmaz, A., Ellsworth, P., Hitomi, M., McClure, E., Marusyk, A., Kaznatcheev, A. and Scott, J.G., 2022. Measuring competitive exclusion in non–small cell lung cancer. Science Advances, 8(26), p.eabm7212.
3. Kaznatcheev, A., Peacock, J., Basanta, D., Marusyk, A. and Scott, J.G., 2019. Fibroblasts and alectinib switch the evolutionary games played by non-small cell lung cancer. Nature ecology & evolution, 3(3), pp.450-456.

To our knowledge, this work will represent the first published drug screen where both **drug synergy** and **cell-cell interactions** are considered and quantified in combination therapy for cancer.

Additionally, the primary aim of our study is not to identify disulfiram as a new drug candidate, but rather to develop and validate a novel framework for identifying and optimizing effective anti-cancer drug combinations, especially in the context of acquired chemoresistance. Our work leverages an integrated experimental-mathematical framework to quantitatively identify disulfiram as an agent that is preferentially effective in chemotherapy-resistant populations, exploiting ecological and evolutionary dynamics within heterogeneous tumors. This application is both novel and timely, given the building interest and clinical need for adaptive, resistance-informed treatment strategies in metastatic ER+ breast cancer.

24th Oct 2025

Manuscript Number: MSB-2025-12854RR
Title: A novel therapy for ER+ breast cancer suppresses drug resistance via an evolutionary double-bind

Dear Dr. Bild,

Thank you for the submission of your revised manuscript to Molecular Systems Biology. We have now received the enclosed report from the referee that was asked to re-assess it for not only their own concerns but also the concerns of the other reviewers. Based on their advice, I am pleased to inform you that we will be able to accept your manuscript pending the following final amendments:

1) Please check the "Author Checklist" carefully and complete all relevant questions. Currently a response for whether computational models are involved in the study is missing.
2) Please ensure that the email address for Dr. Rena Remond is functional. The current address (remond@coh.org) bounces back emails. We would suggest making the secondary email address the primary email address (renaemond@gmail.com).
3) In the main manuscript file, please move the keywords to below the Abstract.
4) Please include the following in the Data Availability statement: 'This study includes no data deposited in external repositories.'
5) Please rename "Declaration of Interests" to "Disclosure and competing interests statement". We updated our journal's competing interests policy in January 2022 and request authors to consider both actual and perceived competing interests. Please review the policy https://www.embopress.org/competing-interests and update your competing interests if necessary.
6) Author contributions: Please remove it from the manuscript and specify author contributions in our submission system. CRediT has replaced the traditional author contributions section because it offers a systematic machine-readable author contributions format that allows for more effective research assessment. You are encouraged to use the free text boxes beneath each contributing author's name to add specific details on the author's contribution. More information is available in our guide to authors:
https://www.embopress.org/page/journal/17574684/authorguide#authorshipguidelines
7) Please correct the reference citation in the reference list to be alphabetical (not numerical). Where there are more than 10 authors on a paper, only the first 10 should be listed, followed by "et al.". Please check "Author Guidelines" for more information.
https://www.embopress.org/page/journal/17574684/authorguide#referencesformat
8) Our journal encourages inclusion of *data citations in the reference list* to directly cite datasets that were re-used and obtained from public databases. Data citations in the article text are distinct from normal bibliographical citations and should directly link to the database records from which the data can be accessed. In the main text, data citations are formatted as follows: "Data ref: Smith et al, 2001" or "Data ref: NCBI Sequence Read Archive PRJNA342805, 2017". In the Reference list, data citations must be labeled with "[DATASET]". A data reference must provide the database name, accession number/identifiers and a resolvable link to the landing page from which the data can be accessed at the end of the reference. Further instructions are available at .
9) Please ensure that a statement on whether or not blinding was done is included in the Methods even if no blinding was done. Please also be sure to update the Author Checklist with this information and where it can be found in the manuscript.
10) Please place individual sections of the manuscript in the following order: Title page - Abstract & Keywords - Introduction - Results - Discussion - Methods - Data Availability - Acknowledgements - Disclosure and Competing Interests Statement - References - Figure Legends - Expanded View Figure Legends.
11) For the figures and figure legends, please take care of the following:
- All figure callouts should be listed sequentially. Currently callouts are missing for Fig. 5D-F. In addition, callouts should be included for the individual panels of EV figures, and the panels should also be referenced in the EV figure legends (i.e. Figure EV1 legend needs to be broken down into panels A-H and referenced so in the main manuscript).
- The EV figures should not be compiled in one file, but uploaded as individual, high-resolution Figure files. The legends should not be included in the EV file, but rather remain in the main manuscript.
- Please note that information related to n is missing in the legends of figures 1B, 4A-C.
- Please note that the error bars are not defined in the legends of figures 1B, 4A-C.
12) The legend for Table 1 should be removed from the manuscript and placed above the table.
13) Synopsis:
- Synopsis image: Please provide a graphic that summarises the main findings of the manuscript on a glance and upload it as a high-resolution jpeg file 550 pixels wide x (300-600) pixels high.
- Synopsis text: Please shorten the standfirst to a maximum of 300 characters, including spaces.
- Please check your synopsis text and image before submission with your revised manuscript. Please be aware that in the proof stage minor corrections only are allowed (e.g., typos).
14) Please either rename each file within each Source Data folder so that it is clear which figure panel the file is associated with or create a subfolder within each Source Data folder with the panel name. Source Data for Figures 3 and 4 should not be in the same zipped folder.
15) As part of the EMBO Publications transparent editorial process initiative (see our policy here: https://www.embopress.org/transparent-process#Review_Process), Molecular Systems Biology will publish online a Peer

Review File (PRF) to accompany accepted manuscripts. This file will be published in conjunction with your paper and will include the anonymous referee reports, your point-by-point response and all pertinent correspondence relating to the manuscript. Let us know whether you agree with the publication of the PRF and as here, if you want to remove or not any figures from it prior to publication. Please note that the Authors checklist will be published at the end of the PRF.
16) After your paper is published, we may promote it on social media. If you have any handles or hashtags for Bluesky you would like included, please let us know.
17) Please provide a point-by-point letter INCLUDING my comments and your detailed responses (as Word file).

I look forward to reading a new revised version of your manuscript as soon as possible.

Yours sincerely,

Poonam Bheda, PhD
Scientific Editor
Molecular Systems Biology

Reviewer #3:

I feel that my concerns have been adequately addressed.

Re: *Manuscript MSB-2025-12854RR*

Title: *A novel therapy for ER+ breast cancer suppresses drug resistance via an evolutionary double-bind*

Date: *December 3, 2025*

Dear Dr. Bheda,

We thank Molecular Systems Biology for accepting our revised manuscript and for providing this final set of comments. We greatly appreciate the careful evaluation by the editor and reviewers and have addressed all requested items in full. Our detailed, point-by-point responses follow below.

1) **Comment**:

"Please check the 'Author Checklist' carefully and complete all relevant questions. Currently a response for whether computational models are involved in the study is missing."

Response:

We appreciate the reminder to review the checklist thoroughly. The Author Checklist has now been fully completed, including the previously omitted item regarding the use of computational models in the study. All associated fields have been reviewed for accuracy and completeness.

2) **Comment**:

"Please ensure that the email address for Dr. Rena Remond is functional. The current address (remond@coh.org) bounces back emails. We would suggest making the secondary email address the primary email address (renaemond@gmail.com)."

Response:

Thank you for alerting us to this issue. We have verified that the original institutional email is no longer active and have updated the submission system accordingly. Dr. Emond's primary contact email is now set to her functional address (renaemond@gmail.com), ensuring that the journal can reliably reach her moving forward.

3) **Comment**:

“In the main manuscript file, please move the keywords to below the Abstract.”

Response:

The keywords have been relocated to immediately follow the Abstract in the main manuscript file, in accordance with the journal’s formatting guidelines. The updated ordering now reflects the standard format used by Molecular Systems Biology.

4) **Comment**:

“Please include the following in the Data Availability statement: 'This study includes no data deposited in external repositories.'”

Response:

Because this study does include computational code and datasets generated as part of the research, we prepared an updated Data Availability statement that transparently reflects the deposited materials. The manuscript now states:

‘All original datasets produced and involved in results and conclusion are provided as source data with this manuscript. All code and data associated with this manuscript will be made publicly available at https://github.com/MathOnco/ER-breast-cancer-evolutionary-double-bind.’

This wording aligns with the journal requirements while accurately describing our data-sharing approach.

5) **Comment**:

“Please rename ‘Declaration of Interests’ to ‘Disclosure and competing interests statement’....”

Response:

This section has been updated to use the correct title, ‘Disclosure and competing interests statement’. We have also reviewed the policy linked in your comment and confirmed that the disclosures provided remain accurate and appropriate under both actual and perceived competing-interest considerations.

6) **Comment**:

“Author contributions: Please remove it from the manuscript and specify author contributions in our submission system....”

Response:

In accordance with journal policy, the Author Contributions section has been removed from the manuscript text. All author contributions have now been entered into the CRediT taxonomy fields within the submission portal, using the free-text boxes to provide more detail. We appreciate the clarity and standardization this system provides.

7) **Comment**:

“Please correct the reference citation in the reference list to be alphabetical (not numerical)....”

Response:

The reference list has been reorganized alphabetically and revised to conform to all journal formatting requirements. Papers with more than 10 authors have been truncated appropriately with ‘et al.’ The entire reference section has been rechecked to ensure internal consistency and compliance with formatting guidelines.

8) **Comment**:

“Our journal encourages inclusion of data citations....”

Response:

We have reviewed this recommendation carefully. Because the present study does not make use of previously published external datasets, data citations are not applicable to this manuscript. No external database records were reused, so no corresponding data references need to be added.

9) **Comment**:

“Please ensure that a statement on whether or not blinding was done is included in the Methods...”

Response:

A clear statement noting that no blinding was performed has now been added to the Methods section. The Author Checklist has also been updated to indicate where this

information appears in the manuscript. This addition ensures transparency regarding study design and analysis.

10) **Comment**:

"Please place individual sections of the manuscript in the following order...."

Response:

The manuscript has been fully reordered to match the structure specified: Title Page → Abstract & Keywords → Introduction → Results → Discussion → Methods → Data Availability → Acknowledgements → Disclosure and Competing Interests Statement → References → Figure Legends → Expanded View Figure Legends. The final document has been reviewed to ensure section continuity and formatting consistency.

11) **Comment**:

"For the figures and figure legends, please take care of the following: …"

Response:

All requested corrections have been carefully implemented. Figure callouts have been checked sequentially, with the missing callouts for Fig. 5D–F now included. Expanded View (EV) figure panels are now referenced individually in both the main text and the EV figure legends, with legends organized by panel (e.g., A–H). EV figures have been separated into individual high-resolution PDF files rather than a compiled set. Missing information regarding sample size (n) and definitions of error bars for Figures 1B and 4A–C has been added. The entire figure set has been systematically reviewed to ensure full compliance.

12) **Comment**:

"The legend for Table 1 should be removed from the manuscript and placed above the table."

Response:

This correction has been completed. The legend is now positioned directly above Table 1 in accordance with the journal's table formatting standards.

13) **Comment**:

“Synopsis: image and text…”

Response:

All requested synopsis components have been updated. A high-resolution graphic (550 px × 300–600 px) summarizing the manuscript’s principal findings has been prepared and uploaded as a separate file. The standfirst will now be shortened to exactly 300 characters and should read:

‘Evolutionary modeling of spheroids composed of sensitive and resistant cells show combination chemo+disulfiram treatment reduces resistance more than sequential therapy, predicting dose-dependent resensitization in vitro.’

Both the synopsis text and image have been reviewed for accuracy prior to submission.

14) **Comment**:

“Please either rename each file within each Source Data folder….”

Response:

All Source Data files have been reorganized so that each dataset clearly reflects the corresponding figure panel. Files have been renamed to map explicitly to each panel, and separate folders have been created where needed. Source Data for Figures 3 and 4 are now correctly separated into different zipped directories.

15) **Comment**:

“As part of the EMBO Publications transparent editorial process initiative…PRF….”

Response:

We agree to publication of the Peer Review File under the EMBO transparent editorial process. We do not request removal of any figures from the PRF. We also acknowledge that the final Authors Checklist will be included in the published PRF.

16) **Comment**:

“After your paper is published, we may promote it on social media…Bluesky….”

Response:

At this time, none of the authors maintain Bluesky accounts or specific hashtags. Should this change in the future, we would be happy to provide updated information for promotional purposes.

17) **Comment**:

“Please provide a point-by-point letter INCLUDING my comments and your detailed responses (as Word file).”

Response:

This document serves as the authors’ detailed point-by-point response to each of the editor’s comments. This has been uploaded as a Word file along with the final submission files.

We again thank the editor and reviewers for their time, guidance, and support throughout this process. Please do not hesitate to contact us should any additional questions arise or if further adjustments are needed.

Sincerely,

Rena Emond

(on behalf of all authors)

13th Jan 2026
Manuscript number: MSB-2025-12854RRR
Title: A novel therapy for ER+ breast cancer suppresses drug resistance via an evolutionary double-bind

Dear Dr. Bild,

Thank you again for sending us your revised manuscript. We are now satisfied with the modifications made and I am pleased to inform you that your paper has been accepted for publication.

Your manuscript will be processed for publication by EMBO Press. It will be copy edited and you will receive page proofs prior to publication. Please note that you will be contacted by Springer Nature Author Services to complete licensing and payment information.

You may qualify for financial assistance for your publication charges - either via a Springer Nature fully open access agreement or an EMBO initiative. Check your eligibility: https://link.springer.com/journal/44320/how-to-publish-with-us

Should you be planning a Press Release on your article, please get in contact with embo_production@springernature.com as early as possible in order to coordinate publication and release dates.

If you have any questions, please do not hesitate to contact the Editorial Office. Thank you for your contribution to Molecular Systems Biology.

Yours sincerely,

Poonam Bheda, PhD
Scientific Editor
Molecular Systems Biology

>>> Please note that it is Molecular Systems Biology policy for the transcript of the editorial process (containing referee reports and your response letter) to be published as an online supplement to each paper. If you do NOT want this, you will need to inform the Editorial Office via email immediately. More information is available here: https://link.springer.com/partners/embo-press/editorial-policies#Peer%20review

EMBO Press Author Checklist

Corresponding Author Name: Rena Emond
Journal Submitted to: Molecular Systems Biology
Manuscript Number: MSB-2025-12854R-Q

USEFUL LINKS FOR COMPLETING THIS FORM
The EMBO Journal - Author Guidelines
EMBO Reports - Author Guidelines
Molecular Systems Biology - Author Guidelines
EMBO Molecular Medicine - Author Guidelines

Reporting Checklist for Life Science Articles (updated January
This checklist is adapted from Materials Design Analysis Reporting (MDAR) Checklist for Authors. MDAR establishes a minimum set of requirements in transparent reporting in the life sciences (see Statement of Task: 10.31222/osf.io/9sm4x). Please follow the journal's guidelines in preparing your
Please note that a copy of this checklist will be published alongside your article.

Abridged guidelines for figures
1. Data
The data shown in figures should satisfy the following conditions:
- the data were obtained and processed according to the field's best practice and are presented to reflect the results of the experiments in an accurate and unbiased manner.
- ideally, figure panels should include only measurements that are directly comparable to each other and obtained with the same assay.
- plots include clearly labeled error bars for independent experiments and sample sizes. Unless justified, error bars should not be shown for technical
- if n<5, the individual data points from each experiment should be plotted. Any statistical test employed should be justified.
- Source Data should be included to report the data underlying figures according to the guidelines set out in the authorship guidelines on Data

2. Captions
Each figure caption should contain the following information, for each panel where they are relevant:
- a specification of the experimental system investigated (eg cell line, species name).
- the assay(s) and method(s) used to carry out the reported observations and measurements.
- an explicit mention of the biological and chemical entity(ies) that are being measured.
- an explicit mention of the biological and chemical entity(ies) that are altered/varied/perturbed in a controlled manner.
- the exact sample size (n) for each experimental group/condition, given as a number, not a range;
- a description of the sample collection allowing the reader to understand whether the samples represent technical or biological replicates (including how many animals, litters, cultures, etc.).
- a statement of how many times the experiment shown was independently replicated in the laboratory.
- definitions of statistical methods and measures:
  - common tests, such as t-test (please specify whether paired vs. unpaired), simple χ2 tests, Wilcoxon and Mann-Whitney tests, can be unambiguously identified by name only, but more complex techniques should be described in the methods section;
  - are tests one-sided or two-sided?
  - are there adjustments for multiple comparisons?
  - exact statistical test results, e.g., P values = x but not P values < x;
  - definition of 'center values' as median or average;
  - definition of error bars as s.d. or s.e.m.

Please complete ALL of the questions below.
Select "Not Applicable" only when the requested information is not relevant for your study.

Materials

**Newly Created Materials**	**Information included in the manuscript?**	**In which section is the information available?** (Reagents and Tools Table, Materials and Methods, Figures, Data Availability Section)
New materials and reagents need to be available; do any restrictions apply?	Not Applicable	

**Antibodies**	**Information included in the manuscript?**	**In which section is the information available?** (Reagents and Tools Table, Materials and Methods, Figures, Data Availability Section)
For **antibodies** provide the following information:<br>- Commercial antibodies: RRID (if possible) or supplier name, catalogue number and or/clone number<br>- Non-commercial: RRID or citation	Not Applicable	

**DNA and RNA sequences**	**Information included in the manuscript?**	**In which section is the information available?** (Reagents and Tools Table, Materials and Methods, Figures, Data Availability Section)
**Short novel DNA or RNA including primers, probes**: provide the sequences.	Not Applicable	

**Cell materials**	**Information included in the manuscript?**	**In which section is the information available?** (Reagents and Tools Table, Materials and Methods, Figures, Data Availability Section)
**Cell lines:** Provide species information, strain. Provide accession number in repository **OR** supplier name, catalog number, clone number, and/**OR** RRID.	Yes	Reagents and Tools Table; Materials and Methods
**Primary cultures:** Provide species, strain, sex of origin, genetic modification status.	Not Applicable	
Report if the cell lines were recently **authenticated** (e.g., by STR profiling) and tested for mycoplasma contamination.	Yes	Materials & Methods

**Experimental animals**	**Information included in the manuscript?**	**In which section is the information available?** (Reagents and Tools Table, Materials and Methods, Figures, Data Availability Section)
**Laboratory animals or Model organisms:** Provide species, strain, sex, age, genetic modification status. Provide accession number in repository **OR** supplier name, catalog number, clone number, **OR** RRID.	Not Applicable	
**Animal observed in or captured from the field:** Provide species, sex, and age where possible.	Not Applicable	
Please detail **housing and husbandry conditions**.	Not Applicable	

**Plants and microbes**	**Information included in the manuscript?**	**In which section is the information available?** (Reagents and Tools Table, Materials and Methods, Figures, Data Availability Section)
**Plants:** provide species and strain, ecotype and cultivar where relevant, unique accession number if available, and source (including location for collected wild specimens).	Not Applicable	
**Microbes:** provide species and strain, unique accession number if available, and source.	Not Applicable	

**Human research participants**	**Information included in the manuscript?**	**In which section is the information available?** (Reagents and Tools Table, Materials and Methods, Figures, Data Availability Section)
If collected and within the bounds of privacy constraints report on age, sex and gender or ethnicity for all study participants.	Not Applicable	

**Core facilities**	**Information included in the manuscript?**	**In which section is the information available?** (Reagents and Tools Table, Materials and Methods, Figures, Data Availability Section)
If your work benefited from core facilities, was their service mentioned in the acknowledgments section?	Not Applicable	

Design

Study protocol	Information included in the manuscript?	In which section is the information available? (Reagents and Tools Table, Materials and Methods, Figures, Data Availability Section)
If study protocol has been **pre-registered, provide DOI in the manuscript**. For clinical trials, provide the trial registration number **OR** cite DOI.	Not Applicable	
Report the **clinical trial registration number** (at ClinicalTrials.gov or equivalent), where applicable.	Not Applicable	

Laboratory protocol	Information included in the manuscript?	In which section is the information available? (Reagents and Tools Table, Materials and Methods, Figures, Data Availability Section)
Provide DOI OR other citation details if **external detailed step-by-step protocols** are available.	Not Applicable	

Experimental study design and statistics	Information included in the manuscript?	In which section is the information available? (Reagents and Tools Table, Materials and Methods, Figures, Data Availability Section)
Include a statement about **sample size** estimate even if no statistical methods were used.	Yes	Materials and Methods; Figures
Were any steps taken to minimize the effects of subjective bias when allocating animals/samples to treatment (e.g. **randomization procedure**)? If yes, have they been described?	Not Applicable	
Include a statement about **blinding** even if no blinding was done.	Yes	Materials and Methods
Describe **inclusion/exclusion criteria** if samples or animals were excluded from the analysis. Were the criteria pre-established?<br><br>If sample or data points were omitted from analysis, report if this was due to attrition or intentional exclusion and provide justification.	Not Applicable	
For every figure, are **statistical tests** justified as appropriate? Do the data meet the assumptions of the tests (e.g., normal distribution)? Describe any methods used to assess it. Is there an estimate of variation within each group of data? Is the variance similar between the groups that are being statistically compared?	Yes	Materials and Methods; Figures

Sample definition and in-laboratory replication	Information included in the manuscript?	In which section is the information available? (Reagents and Tools Table, Materials and Methods, Figures, Data Availability Section)
In the figure legends: state number of times the experiment was **replicated** in laboratory.	Yes	Figures; Materials and Methods
In the figure legends: define whether data describe **technical or biological replicates**.	Yes	Figures; Materials and Methods

Ethics

Ethics	Information included in the manuscript?	In which section is the information available? (Reagents and Tools Table, Materials and Methods, Figures, Data Availability Section)
Studies involving **human participants**: State details of **authority granting ethics approval** (IRB or equivalent committee(s), provide reference number for approval.	Not Applicable	
Studies involving **human participants**: Include a statement confirming that **informed consent** was obtained from all subjects and that the experiments conformed to the principles set out in the WMA Declaration of Helsinki and the Department of Health and Human Services Belmont Report.	Not Applicable	
Studies involving **human participants:** For publication of **patient photos**, include a statement confirming that consent to publish was obtained.	Not Applicable	
Studies involving experimental **animals**: State details of **authority granting ethics approval** (IRB or equivalent committee(s), provide reference number for approval. Include a statement of compliance with ethical regulations.	Not Applicable	
Studies involving **specimen and field samples:** State if relevant **permits** obtained, provide details of authority approving study; if none were required, explain why.	Not Applicable	

Dual Use Research of Concern (DURC)	Information included in the manuscript?	In which section is the information available? (Reagents and Tools Table, Materials and Methods, Figures, Data Availability Section)
Could your study fall under dual use research restrictions? Please check biosecurity documents and list of **select agents and toxins** (CDC): https://www.selectagents.gov/sat/list.htm	Not Applicable	
If you used a select agent, is the security level of the lab appropriate and reported in the manuscript?	Not Applicable	
If a study is subject to dual use research of concern regulations, is the name of the **authority granting approval and reference number** for the regulatory approval provided in the manuscript?	Not Applicable	

Reporting

The MDAR framework recommends adoption of discipline-specific guidelines, established and endorsed through community initiatives. Journals have their own policy about requiring specific guidelines and recommendations to complement MDAR.

Adherence to community standards	Information included in the manuscript?	In which section is the information available? (Reagents and Tools Table, Materials and Methods, Figures, Data Availability Section)
State if relevant guidelines or checklists (e.g., **ICMJE, MIBBI, ARRIVE, PRISMA**) have been followed or provided.	Not Applicable	
For **tumor marker prognostic studies,** we recommend that you follow the **REMARK** reporting guidelines (see link list at top right). See author guidelines, under 'Reporting Guidelines'. Please confirm you have followed these guidelines.	Not Applicable	
For **phase II and III randomized controlled trials**, please refer to the **CONSORT** flow diagram (see link list at top right) and submit the CONSORT checklist (see link list at top right) with your submission. See author guidelines, under 'Reporting Guidelines'. Please confirm you have submitted this list.	Not Applicable	

Data Availability

Data availability	Information included in the manuscript?	In which section is the information available? (Reagents and Tools Table, Materials and Methods, Figures, Data Availability Section)
Have **primary datasets** been deposited according to the journal's guidelines (see 'Data Deposition' section) and the respective accession numbers provided in the Data Availability Section?	Yes	Will be Data Availability Section
Were **human clinical and genomic datasets** deposited in a public access-controlled repository in accordance to ethical obligations to the patients and to the applicable consent agreement?	Not Applicable	
Are **computational models** that are central and integral to a study available without restrictions in a machine-readable form? Were the relevant accession numbers or links provided?	Yes	Data availability section; All code associated with this manuscript will be made publicly available at https://github.com/MathOnco/ER-breast-cancer-evolutionary-double-bind.
If publicly available data were reused, provide the respective **data citations in the reference list**.	Not Applicable	
